# tRNA Fragments Populations Analysis in Mutants Affecting tRNAs Processing and tRNA Methylation

**DOI:** 10.3389/fgene.2020.518949

**Published:** 2020-10-09

**Authors:** Anahi Molla-Herman, Margarita T. Angelova, Maud Ginestet, Clément Carré, Christophe Antoniewski, Jean-René Huynh

**Affiliations:** ^1^Collège de France, CIRB, CNRS Inserm UMR 7241, PSL Research University, Paris, France; ^2^Transgenerational Epigenetics & Small RNA Biology, Sorbonne Université, CNRS, Laboratoire de Biologie du Développement - Institut de Biologie Paris Seine, Paris, France; ^3^ARTbio Bioinformatics Analysis Facility, Sorbonne Université, CNRS, Institut de Biologie Paris Seine, Paris, France

**Keywords:** *Drosophila*, Nm methylation, RNase P, tRNA, tRFs, oogenesis

## Abstract

tRNA fragments (tRFs) are a class of small non-coding RNAs (sncRNAs) derived from tRNAs. tRFs are highly abundant in many cell types including stem cells and cancer cells, and are found in all domains of life. Beyond translation control, tRFs have several functions ranging from transposon silencing to cell proliferation control. However, the analysis of tRFs presents specific challenges and their biogenesis is not well understood. They are very heterogeneous and highly modified by numerous post-transcriptional modifications. Here we describe a bioinformatic pipeline (tRFs-Galaxy) to study tRFs populations and shed light onto tRNA fragments biogenesis in *Drosophila melanogaster*. Indeed, we used small RNAs Illumina sequencing datasets extracted from wild type and mutant ovaries affecting two different highly conserved steps of tRNA biogenesis: 5′pre-tRNA processing (RNase-P subunit Rpp30) and tRNA 2′-O-methylation (dTrm7_34 and dTrm7_32). Using our pipeline, we show how defects in tRNA biogenesis affect nuclear and mitochondrial tRFs populations and other small non-coding RNAs biogenesis, such as small nucleolar RNAs (snoRNAs). This tRF analysis workflow will advance the current understanding of tRFs biogenesis, which is crucial to better comprehend tRFs roles and their implication in human pathology.

## Introduction

Transfer RNAs (tRNAs) are molecules of ∼75 nt transcribed by RNA polymerase III that adopt a typical cloverleaf secondary structure. They are ancient molecules required for protein translation and are encoded by hundreds of genes (∼300 in *Drosophila*, ∼400 in humans) localized in clusters throughout the genome in some species (Haeusler and Engelke, 2006; Willis and Moir, 2018). tRNAs can be transcribed in the nucleus or in mitochondria. Once transcribed, tRNA precursors (pre-tRNAs, ∼125 nt) are processed by the highly conserved ribozymes RNAse P and Z, to cleave the 5′ leader and the 3′ trailer, respectively (Jarrous, 2017). Then, a CCA trinucleotide tag is added at the 3′ end of mature tRNAs by a specific enzyme (RNA polymerase ATP(CTP):tRNA nucleotidyltransferase) present in all kingdoms of life. CCA tag plays a role in tRNA amino-acylation, tRNA export toward the cytoplasm, and tRNA quality control (Wellner et al., 2018). RNase P is formed by one RNA molecule and several protein subunits such as Rpp30, highly conserved throughout evolution (Jarrous, 2017). In some species, RNAse P can also cleave non-canonical targets such as rRNA, snoRNA, some long non-coding RNA and RNAs containing N6-methyladenosine (m^6^A) (Coughlin et al., 2008; Jarrous, 2017; Park et al., 2019).

Importantly, tRNA biogenesis involves the production of small RNA molecules, hereafter referred to as tRNA fragments (tRFs), derived either from tRNA precursors or from cleavage of mature tRNAs. tRFs are found in a wide variety of organisms and tissues and are associated with several pathologies such as cancer and neurodegeneration (reviewed Kumar et al., 2016; Soares and Santos, 2017; Shen et al., 2018). Despite recent efforts to develop tools describing tRFs populations (Thompson et al., 2008; Kumar et al., 2014b; Selitsky and Sethupathy, 2015; Pliatsika et al., 2016; Loher et al., 2017a; Schorn et al., 2017; Kuscu et al., 2018; Liu et al., 2018; Guan et al., 2019) tRFs analyses from different laboratories remain difficult to compare (Supplementary Table 1). Indeed, finding consensus tools to study different species and tissues is difficult for several reasons (Telonis et al., 2016). First, different factors can vary in RNA sample preparation (protocol, tissue, species, sex, population…) as well as in library preparation. Secondly, tRFs nomenclature, bioinformatics workflows, bioinformatics softwares and parameters vary depending on the laboratory. Thirdly, tRNAs-genome references are different in each species^1^ and their construction to get all tRFs types can vary depending on the study. Fourthly, it has been suggested that very small RNAs (14–16 nt) could originate not only from tRNA molecules, but also from highly repeated regions unrelated with tRNA, or from incomplete (truncated) pseudo-tRNAs in some organisms, with different copy numbers and genomic localizations (Telonis et al., 2014). Also, tRNAs can be substrates for the production of other types of small ncRNA such as miRNAs or piRNAs (Maute et al., 2013; Keam et al., 2014; Honda et al., 2017). This problem can be addressed by studying tRFs that match the “non-tRNA-space,” which corresponds to the whole genome excluding tRNA genes (Telonis et al., 2016; Loher et al., 2017b). Importantly, while trying to exclude false positive tRFs, one could increase false negative error rate, since it is difficult to know the real origin of tRFs: “tRNA space,” “non-tRNA space,” or both. In addition, some nuclear tRNAs can be similar to mitochondrial tRNAs in vertebrates (especially in primates). These tRNAs, called tRNA-lookalikes, could be a source of tRFs, whose origin is difficult to determine. However, no tRNAs-lookalike were found in *Drosophila* using perfect match alignments, and only one tRNA-lookalike was found allowing mismatches (Telonis et al., 2014, 2015a). Finally, several tRNAs corresponding to the same amino-acid share the same sequence^2^. Thus, these tRNAs will generate different types of tRFs which can be attributed randomly to one of these tRNAs or to all of them (ex.tRNA:Val-CAC-2-1 to 2-6). This problem can be solved by collapsing tRNA sequences to obtain unique tRNA mature sequences. However, this collapse cannot be done with the extended sequences of tRNAs (25 nt and 80 nt flanking mature tRNA) since these sequences are different. Besides, only some bioinformatic analysis have tried to validate tRFs profiles in parallel, by performing Northern Blot (Torres et al., 2019) (Supplementary Table 1).

The impact of tRFs levels in various biological processes is currently under investigation and multiple processes have already been identified, amongst which stands gene expression and translation control, transposon silencing, ncRNA processing, histone levels control, cell proliferation and DNA damage response modulation (Goodarzi et al., 2015; Sharma et al., 2016, 2018; Kuscu et al., 2018; Li et al., 2018; Liu et al., 2018; Schorn and Martienssen, 2018; Shen et al., 2018; Boskovic et al., 2019; Guan et al., 2019; Su et al., 2019).

**FIGURE 1:**
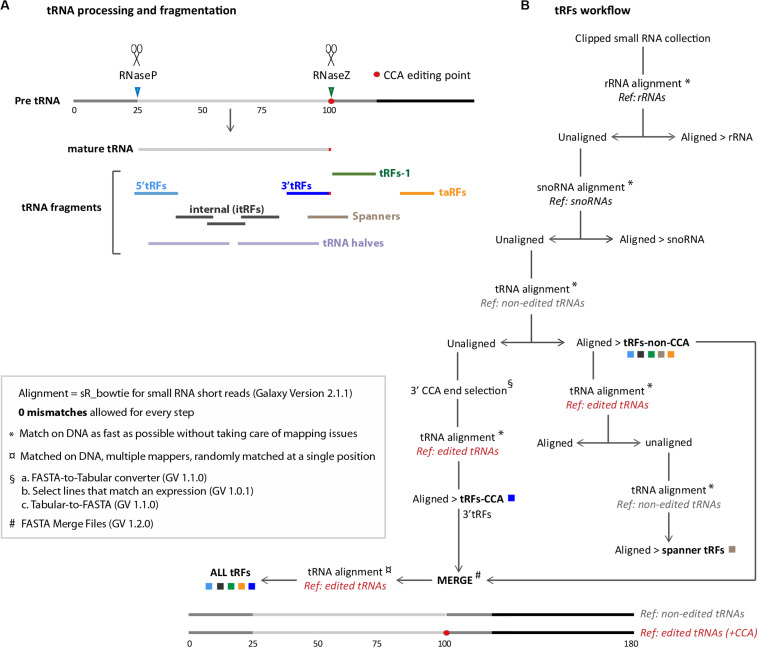
General workflow for tRNA fragments (tRFs) classes extraction: **(A)** tRNA processing and tRNA fragments are depicted. The 5′ tail of pre-tRNAs is cleaved by RNase P (blue arrowhead) and the 3′ tail is cleaved by RNase Z (green arrowhead). 5′ cleavage product is believed to be degraded whereas RNase Z cleavage product forms tRFs-1 (green line). Mature tRNAs (light gray line) is edited by the addition of 3′-tRFs motif (red dot). Several types of tRFs can be generated from mature RNAs, such as 5′-tRFs (light blue line), 3′-tRFs (dark blue line), and inner tRFs (i-tRFs) belonging to the anticodon region (dark gray lines). Spanner-tRFs can be formed before the addition of CCA from tRNA-precursors, spanning the CCA region (light brown line). Transcription associated (taRFs, orange line) can be formed from downstream regions of tRNAs. Longer tRNA halves are represented with light purple lines. **(B)** Galaxy-developed workflow for extraction of all tRFs classes, described in **A**. Alignments were done with SR_Bowtie tool for small RNA short reads (version 2.1.1) using two types of matching: ^∗^ Match on DNA as fast as possible or ¤ Match on DNA, multiple mappers. “*Ref.”* are the different genome references used for alignments in this pipeline: rRNA, snoRNA, tRNA-non-edited or tRNA-CCA-edited. For tRNA-non-edited reference construction, mature tRNAs (75 nt) were compared with tRNA-precursors (125 nt) to determine RNase P and RNase Z cleavage points. 25 nt were added upstream at 5′, and 80 nt downstream, right after the RNase Z cleavage point (25 + 75 + 80 = 180 nt approximately). For tRNA-CCA-edited reference construction, a CCA motif was added to the non-edited reference, precisely at the 3′CCA edition point (red dot). tRFs CCA or non-CCA can be treated separately or altogether (ALL-tRFs).

In wild type condition, when RNAse P cleaves the 5′ trailer of tRNA-precursor, the resulting fragment is believed to be degraded by the ribonuclease translin–TRAX complex (C3PO) (Li Z. et al., 2012; Figure 1A). Then, RNase Z cleaves the 3′ trailer forming tRFs-1 (also called tRFs-3′U because the Poly-U tract is typically found at 3′ of pre-tRNAs) (Rossmanith, 2012; Kumar et al., 2015). Once mature, tRNAs can be cleaved forming small fragments: tRFs-5 (or 5′tRFs, originating from 5′) or tRFs-3 (or 3′tRFs, originating from 3′ including CCA tag). These cleavages could be done by Dicer or by other endonucleases that remain to be discovered (Cole et al., 2009; Sobala and Hutvagner, 2011; Li L. et al., 2012; Kuscu et al., 2018; Shen et al., 2018; Su et al., 2019). Internal tRFs (i-tRFs) are contained to the interior of the mature tRNA sequence and can straddle the anticodon (Telonis et al., 2015b). Also, mature tRNA molecules can be cut in 2 halves (tRNA halves ∼35 nt) which play important roles in different stress conditions, such as hypoxia or temperature changes (Fu et al., 2008; Thompson et al., 2008; Shen et al., 2018; Akiyama et al., 2020). Intriguingly, in some neuropathologies, tRNA precursors can be cleaved and generate tRNA fragments (∼40 nt) which include the 5′ trailer (Hanada et al., 2013). Spanner-tRFs are another class of tRFs that occur rarely and can be formed before the RNase Z cleavage and before CCA addition, spanning the CCA editing point. Finally, transcription termination associated tRNA fragments (taRFs) are formed when RNA Pol-III does not finish transcription properly. Interestingly, altered tRF populations have been discovered in mouse mutants for RNase Z (ELAC2), which have cardiomyopathy and premature death (Siira et al., 2018). However, it is still not known whether RNase P also plays a role in tRFs formation.

Aberrant tRFs populations could have *trans* effects on gene expression. They could target different RNAs by sequence complementarity, by guiding Argonaute proteins similarly to other small non-coding RNAs like miRNAs (microRNAs), siRNAs (small interfering RNAs) and piRNAs (Piwi-interacting RNAs) (Kim et al., 2009; Kumar et al., 2014a; Yamanaka and Siomi, 2015). miRNAs are small RNAs known to cleave mRNAs or inhibit mRNA translation (Jonas and Izaurralde, 2015). piRNAs and siRNAs are small RNAs known to silence transposable elements (TEs) (Czech et al., 2018). Among tRFs targets, some TEs and gene sequences have been identified, linking tRFs to several cellular processes and pathologies, such as translation control, cell signaling, development, proteasome regulation or metabolism (Goodarzi et al., 2015; Karaiskos and Grigoriev, 2016; Sharma et al., 2016; Martinez, 2017; Schorn et al., 2017; Kim et al., 2019; Mo et al., 2019; Telonis et al., 2019). tRFs thus emerge as potential biomarkers and therapeutic targets for human pathologies (Balatti et al., 2017; Zhu et al., 2018).

Currently, around 150–170 RNA modifications are known, and recent reports show that RNA modifications defects play an important role in tRFs production in different organisms. Epitranscriptomics have recently emerged as a new field to comprehend the mechanisms underlying RNA modifications and their role in gene expression. Indeed, tRNAs are the most extensively modified RNAs in cells (up to 25% of nucleotides per tRNA) (Delaunay and Frye, 2019; Ontiveros et al., 2019; Guzzi and Bellodi, 2020). These marks are believed to help tRNAs to respond to a wide range of environmental cues, stimuli and stress. They play crucial roles at all tRNA biogenesis steps, such as sequence maturation, folding, recycling and degradation. Interestingly, there is a crosstalk between the different modification pathways and a large amount of tRNA modification enzymes defects have been linked to human pathologies (Angelova et al., 2018; Sokołowski et al., 2018; Lyons et al., 2018; Dimitrova et al., 2019).

In *Drosophila* it has been recently shown that methylation marks protect tRNAs from cleavage and that aberrant tRFs populations accumulate in methylation mutants: on the one hand, Dnmt2 mutation impairs m^5^C methylation (Schaefer et al., 2010; Durdevic et al., 2013a; Genenncher et al., 2018). On the other hand, dTrm7_34 (CG7009) and dTrm7_32 (CG5220) mutation impairs 2′-O-methylation (Angelova et al., 2020). 2′-O-methylation is one of the most common RNA modifications and consists in the addition of a methyl group to the 2′ hydroxyl of the ribose moiety of a nucleoside, being also known as Nm. It is found in tRNAs, rRNAs, snRNAs (small nuclear RNAs), at the 3′ end of some small non-coding RNAs (such as piRNAs), and at some sites on mRNAs (Ontiveros et al., 2019). This modification plays a wide range of roles in RNA structure, stability and interactions (Dimitrova et al., 2019). It has been recently shown that *Drosophila* proteins dTrm7_34 and dTrm7_32 are the functional orthologs of yeast TRM7 (Pintard, 2002) and human FTSJ1 (Guy et al., 2015) respectively, which are involved in 2′-O-methylation of the anticodon loop of several conserved tRNAs substrates (tRNA-Leu, Trp, Phe). Mutations of these tRNAs methyltransferases in *Drosophila* lead to lifespan reduction, small non-coding RNA pathways dysfunction and increased sensitivity to RNA virus infections, besides specific tRFs accumulation (Angelova et al., 2020).

Despite their abundance, only a very limited subset of RNA modifications can be detected and quantified by current high-throughput analytical techniques such as ARM-seq, and substantial efforts are being invested for the development of this field (Cozen et al., 2015; Dai et al., 2017). Some modifications, such as 2′-O-methylation, can have an impact on classical sequencing techniques during library preparation (reverse transcription blocking) and could introduce a bias in the analyses, such as in the type of tRFs preferentially sequenced which can have different degrees of modification (Motorin and Helm, 2019). However, one study have reported that tRNA modifications only have a limited impact on data mining when studying tRFs in The Cancer Genome Atlas (Telonis et al., 2019). Indeed, we still do not know the impact of each RNA modification on small RNA sequencing, and thousands of small RNA datasets have already been generated with Illumina sequencing techniques. Thus, a wide range of wild type and mutant datasets from different species are available^3^ ^,4^ and their analysis can bring important new information on tRFs biogenesis and/or stability.

Since tRFs biogenesis remains obscure, we developed and describe a user-friendly tRFs-pipeline for *Drosophila melanogaster* based on Galaxy environment (tRFs-Galaxy), with workflows and tools that can be easily shared with the scientific community. To do so, we took advantage of several *Drosophila* datasets (15–29 nt) generated in our laboratories: *Rpp30* mutants, which affect tRNA processing, and dTrm7_34 and dTrm7_32, which affect tRNA Nm methylation (Molla-Herman et al., 2015; Mollà-Herman et al., 2019; Angelova et al., 2020). We believe that this study will help to better understand the known pathways of tRFs biogenesis as well as to uncover new tRFs biogenesis factors and unexpected crosstalks between different RNA regulatory mechanisms, crucial for gene expression.

## Materials and Methods

### Fly Stocks

Fly stocks are described in Molla-Herman et al. (2015) and Angelova et al. (2020).

### RNA Extraction From Ovaries

RNA was extracted from *Drosophila* ovaries following standard methods detailed in Molla-Herman et al. (2015) and Angelova et al. (2020).

### Small RNA Sequencing

RNA samples of 3–5 μg were used for High-throughput sequencing using Illumina HiSeq, 10% single-reads lane 1 × 50 bp (Fasteris). 15–29 nt RNAs sequences excluding rRNA (riboZero) were sequenced. All the analyses were performed with Galaxy tools^5^. Workflows are available upon request. Data set deposition is described in Molla-Herman et al. (2015) and Angelova et al. (2020). European Nucleotide Archive (ENA) of the EMBL-EBI^6^, accession numbers are: PRJEB10569 (Rpp30 mutants), PRJEB35301 and PRJEB35713 (Nm mutants).

### Clipping and Concatenation

Raw data were used for clipping the adaptors [Clip adapter (Galaxy-Version 2.3.0, owner: artbio)] and FASTQ quality control was performed [FastQC Read Quality reports (Galaxy-Version 0.72)]. Since replicates were homogeneous in quality and analysis (replicates for heterozygous and homozygous *dTrm7_34^∗^* flies and triplicates for *dTrm7_34^∗^- dTrm7_32^∗^* double mutants) we merged them [Concatenate multiple datasets tail-to-head (Galaxy-Version 1.4.1, owner: artbio) to have single fasta files. *dTrm7_34^∗^/Def9487 as well as Rpp30^18.2^, mnk^*P*6^ homozygous and Rpp30^*PE*^/Rpp30^18.2^* datasets were used to obtain normalization numbers but are not shown in the figures for simplicity (Supplementary Figure 8).

### Data Normalization Using DeSeq miRNA Counts

Data were normalized with library Normalization Factors (NF) obtained by using [DESeq geometrical normalization (Galaxy-Version 1.0.1, owner: artbio)] with miRNA counts obtained using [miRcounts (Galaxy-Version 1.3.2)], allowing 0 mismatch (MM). Then, 1/NF values were used in Galaxy small RNA maps (Supplementary Figures 8A,B).

### Data Normalization With DeSeq Using tRFs Counts

To create tRFs expression heatmaps, all-tRFs read counts were normalized using [DESeq Normalization (Galaxy-Version 1.0.1, owner: artbio)] giving rise to a Normalized Hit Table.

### Genome References

rRNA, snoRNA, miRNA, ncRNA, intergenic, genic references and Transposable Elements (Ensemble canonical TE) were obtained from Ensembl Biomart^7^. For tRNAs, we created a genome reference of extended pre-tRNAs adding 25 nt upstream and 80 nt downstream of tRNAs genome annotations. These sequences referred to as “non-edited tRNAs” have an average length of ∼180.3 nt (Standard Deviation 14.9 nt) for nuclear tRNAs and ∼170 nt (Standard Deviation 6.2 nt) for mitochondrial tRNAs. Sixteen tRNA sequences have an intron that has to be spliced. To analyze tRFs carrying 3′CCA motif we inserted a CCA in the genomic precursor sequence, at the position where tRNAs are edited after pre-tRNA maturation. We called this reference “CCA-edited-tRNAs.” To study the “non-tRNA space” we created a reference genome excluding known tRNAs gene segments. To avoid multimapping of tRFs to several tRNAs with similar sequences we collapsed tRNAs mature sequences into “Unique Mature tRNAs” and we added CCA tag. We split the snoRNA sequences in two reference sets, one with box C/D snoRNAs whose mature sequences are equal or less than 120 nt long, the other with box H/ACA snoRNAs whose mature sequences are more than 120 nt long.

### General Small RNA Annotation

Small RNA reads files were first depleted from rRNAs by discarding reads aligning to rRNA genome reference. Then, we annotated the small RNAs by iterative alignments to the various references using the tool [Annotate smRNA dataset (Galaxy-Version 2.4.0, owner: artbio)] and allowing 0 mismatches. For annotation cascades, iterative alignments were performed in the following order: tRNA, tRNA-CCA-edited, miRNA, TE-derived, all-ncRNA, all genes and all intergenic. The number of alignments for each class were visualized with Pie-Charts whose sizes reflect the respective depth (total aligned reads) of the libraries (see Supplementary Figure 8C).

### Specific tRFs Classes Extraction

Small RNA reads trimmed off from their adapter sequences were first aligned to the rRNA reference using the Galaxy tool [sR_bowtie (Galaxy-Version 2.1.1, owner: artbio)] and the option “Match on DNA as fast as possible.” Unaligned reads were retrieved and aligned to the snoRNA reference, and snoRNA alignments were visualized using the tool [small RNA maps (Galaxy-Version 2.16.1, owner: artbio)].

Next, unaligned reads were retrieved and realigned to the non-edited tRNA reference. Matching reads in this step correspond to tRFs without CCA (tRF-non-CCA) including 5′-tRFs, tRFs-1, spanners and internal tRFs. On the contrary, edited 3′-tRFs did not match in this step, because the CCA motif is not encoded in the genome and we did not allow mismatches (see below). To retrieve these unmatched tRFs, we selected unaligned reads with 3′ end CCA and realigned these reads to the CCA-edited-tRNA reference.

Finally, we merged non-CCA tRFs and 3′ tRF using the tool [FASTA Merge Files (Galaxy-Version 1.2.0)] and realigned those reads to the CCA-edited-tRNA reference. Matched reads (“all-tRFs”) were visualized (see Figure 1B) using the tool [small RNA maps (Galaxy-Version 2.16.1, owner: artbio)].

In order to isolate spanner tRFs, aligned non-CCA-tRFs were realigned using CCA-edited tRNAs as reference. Unaligned reads in this step are tRFs that span the editing point. These reads were realigned using non-edited-tRNA reference, allowing to retrieve spanner-tRFs maps.

Importantly, we could not reliably detect tRNA Halves (> 30 nt) since our original libraries were prepared using RNA size selection between 15 and 29 nt.

### tRFs Global Size Distribution, Coverage and tRF Logo

All-tRFs, non-CCA-tRFs or 3′-tRFs datasets were used to generate small RNA maps and read size distributions taking into account the normalization factors for the different genotypes. Read coverage of tRNA sequences was generated using the tool [BamCoverage (Galaxy-Version 3.1.2.0.0, owner: bgruening)]. Briefly, we first used sR_bowtie with the options “matched on DNA, multiple mappers randomly matched at a single position,” “0 mismatch allowed,” and tRNA-CCA-edited as a reference. Bam alignment files from this step were used with the BamCoverage tool to generate BigWig coverage files, using the library normalization factors as scale factors. The tool [computeMatrix (Galaxy-Version 3.1.2.0.0, owner: bgruening)] was then used to prepare the data for plotting heatmaps or a profile of given regions. We used four Bed files with this tool to visualize Nuclear tRNAs, 5′-tRFs, 3′-tRFs and Mitochondrial tRNAs (see Supplementary Figure 9A). To obtain a Logo, tRFs FASTA files were treated to obtain the last 15 nt of every sequence then we used the tool [Sequence Logo (Galaxy-Version 3.5.0, owner: devteam)].

### tRFs Expression Heatmap and Ratio Calculation

To visualize tRFs expression levels we created Heatmaps. With all-tRFs collection list, we used sR_Bowtie (for small RNA short reads Galaxy-Version 2.1.1, matched on DNA, multiple mappers, randomly matched at a single position, 0 mismatch allowed) and we used tRNA-CCA-edited as reference. Then we used the tool [Parse items in sR_Bowtie alignment (Galaxy-Version 1.0.6)]. We did a DESeq2 normalization of hit lists (geometrical method Galaxy-Version 1.0.1, see above). We cut columns from the Normalized Hit table (Galaxy-Version 1.0.2) and we used Sort data in ascending or descending order tool (Galaxy-Version 1.0.0), generating a table with the tRFs counts for the different genotypes. We used Plot Heatmap with high number of rows (Galaxy-Version 1.0.0) to create the expression profiles. We used Log2(value + 1) and Blue-White-Red colors to reflect reads from minimal to maximal expression. We created Heatmaps using the “tRNA-extended-CCA-edited” genome of reference to have all types of tRFs represented. This method leads to multimapping issues of several tRFs that match different tRNAs genes with similar sequences. We thus also created Heatmaps using the “Unique tRNA mature CCA-edited” genome of reference that avoids multimapping but leads to the loss of tRFs-1 originating from the precursor. To detect important changes of tRFs between genotypes, we cut columns corresponding to counts of *white*^–^ and *Rpp30^18.2^* mutants, or *dTrm7_34^∗^/TbSb* heterozygous and *dTrm7_34^∗^* homozygous mutants. We calculated the ratio of tRFs expression between them, using Compute an expression on every row tool (Galaxy-Version 1.2.0). The obtained data were treated with Microsoft Office Excel to better observe ratio differences by using conditional formatting tool, obtaining a three color code (Blue-White-Red from minimal to maximal value, see Supplementary Figure 9B).

### snoRNAs Global Size Distribution and Coverage

To represent all the reads along a canonical snoRNA molecule we analyzed the Bam Coverage, by first using sR_Bowtie for small RNA short reads (Galaxy-Version 2.1.1), matched on DNA, multiple mappers, randomly matched at a single position. 0 mismatches were allowed, using snoRNA as genome reference. Then BamCoverage tool generates a coverage BigWig file from a given BAM file (Galaxy-Version 3.1.2.0.0) that we normalized using the scale factors. Afterward, Compute Matrix prepares data for plotting a heatmap or profiles of given regions (Galaxy-Version 3.1.2.0.0). We had three Bed files to plot: snoRNAs > 120 nt Bed file; snoRNAs < 120 nt Bed file; and both together (see Supplementary Figure 9C).

## Results

### How to Study Different tRFs Categories

In this study we have developed user friendly and easy to share workflows using Galaxy^5^ allowing to extract all major classes of tRFs (tRFs-Galaxy) (Figure 1 and see section “MATERIALS AND METHODS”): 5′-tRFs, 3′-tRFs and inner-tRFs, corresponding to fragments derived from mature tRNA transcripts; tRFs-1, formed by RNase-Z cleavage of tRNA precursors; spanner tRFs, spanning the CCA region and created before CCA addition; and transcription associated tRFs (taRFs), formed due to problems in transcription termination. The presented pipeline allows to study them separately or altogether.

### tRFs Description in *Drosophila* Ovaries

**FIGURE 2:**
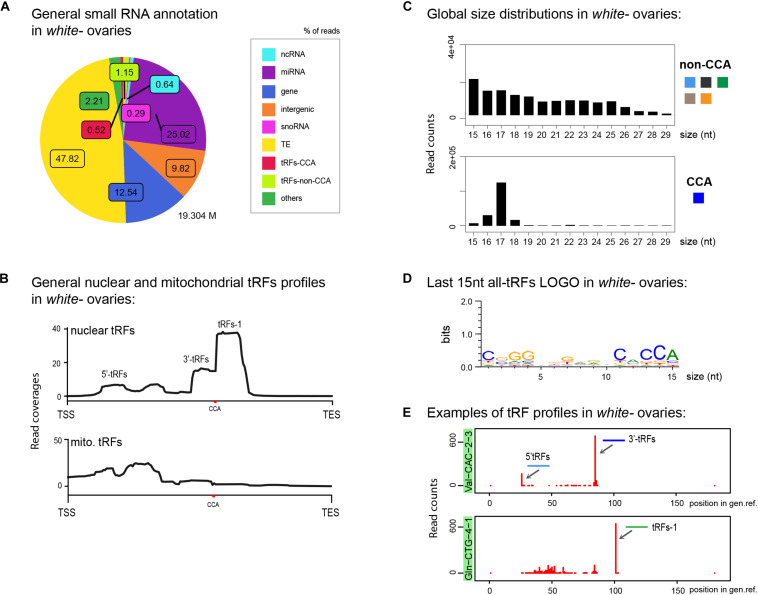
tRFs description in control *Drosophila* ovaries: **(A)** Small RNAs sequences from 15–29 nt were analyzed to distinguish different categories with the help of an annotation cascade tool in the following order: miRNA, ncRNA, intergenic, genes, TE (piRNA, siRNA), snoRNAs, tRFs-non-CCA or tRFs-CCA. The percentage of reads is shown in a pie-chart, which size reflects the bank’s depth (M = Millions of reads). **(B)** Nuclear and mitochondrial tRFs coverages of 15–29 nt tRFs were analyzed in *white-* control ovaries using scaling factors (see section “MATERIALS AND METHODS”). CCA edition point is shown with a red dot. The different types of tRFs are shown along the coverage profile from the beginning of the pre-tRNA molecule (TSS transcription start site) to the end of the extended edited genome reference (TES, transcription extended site). **(C)** General size distribution (15–29 nt) of normalized read counts corresponding to different categories of tRFs in *white-* control ovaries. Color-codes on the right are the same as in Figure 1B for tRFs categories. **(D)** Logo for the last 15 nt of *white-* tRFs sequences (all categories included, issued from *fasta* files). **(E)** Examples of tRFs readmap profiles in *white-* control ovaries originating from two different tRNAs. Red peaks reflect read counts (using scaling factors). The position of the peak along the edited tRNA reference genome reflects the beginning of the reads sequences. 0: beginning of the pre-tRNA. 100: position of RNase Z cleavage. 5′-tRFs are in light blue, 3′-tRFs are in dark blue, tRFs-1 are in green.

To describe tRFs general populations in *wild type* ovaries from young flies, we first performed a cascade of annotations of small RNA populations, to the exclusion of rRNA fragments which were previously depleted from the sequence datasets (Figure 2A and Supplementary Figure 8) (rRNA were “bioinformatically depleted”). A high percentage of small RNA reads correspond to transposable elements (TEs, yellow), representing piRNAs and/or siRNA that match TE sequences. To distinguish tRFs carrying a 3′CCA motif from non-CCA-tRFs (5′-tRFs, i-tRFs, spanners, taRFs and tRFs-1) we used two different reference genome files (see below). In *white*^–^ ovaries there are twice as much non-CCA-tRFs than 3′-tRFs (Figure 2A: 1.15% *vs* 0.52%). However, since some sequences can be matched by multiple types of small ncRNAs, the mapping order in the cascade annotation tool can introduce a bias, as observed in the MINTmap tRFs study of Loher et al. (2017b). Thus, we used different tools to study tRFs populations in detail.

To have a general overview of tRNA fragments, we aligned all tRFs along canonical nuclear or mitochondrial tRNA precursors, belonging to 290 different nuclear tRNAs and 21 different mitochondrial tRNAs (Figure 2B). Nuclear tRFs coverage shows that in *white*^–^ control ovaries there is a majority of tRFs-1. In addition, we observe a significant population of 3′-tRFs and a minor population of 5′-tRFs and inner tRFs. Mitochondrial tRFs seem more abundant at the 5′ part of tRNAs molecules and around the anticodon region. In addition, global size distribution analysis showed that in control ovaries, non-CCA-tRFs are heterogeneous, ranging from 15 to 25 nt, whereas 3′-tRFs are mostly 17 nt long (Figure 2C). The presence of a CCA signature could be easily identified by analyzing the Logo of the last 15 nt of tRFs populations (Figure 2D).

We next interrogated which type of tRNAs molecules could generate these tRFs. In *Drosophila melanogaster* there are 21 mitochondrial tRNAs (one per amino-acid) and 290 nuclear tRNAs, comprising several tRNAs per isotype with different anticodon sequences (between 5 and 22 tRNAs per amino acid)^1^. For example, there are 15 tRNAs for Valine with different anticodons: 6 tRNA:Val-AAC, 7 tRNA:Val-CAC and 2 tRNA:Val-TAC. Among tRNA genes, 16 tRNAs carry an intron (tRNA:Leu-CAA, Ile-TAT and Tyr-GTA). Since tRNA genes are redundant, the physiological importance of expression levels variations of individual tRNA genes is not well understood. However, it has been recently shown that differential tRNA gene expression results in changes in the abundance of tRFs but not of mature tRNAs, suggesting that different expression levels of tRNA genes may regulate non-canonical tRNA functions through tRFs (Torres et al., 2019).

Moreover, it has been shown in some organisms that small tRFs sequences could originate from genome regions similar to tRNAs, which are not true tRNA genes. These regions can be tRNA-lookalikes, truncated tRNA genes or repeated elements and they form the “non-tRNA-space” (Telonis et al., 2016; Loher et al., 2017b) (Supplementary Figure 1A). Thus, it is difficult to know the genomic origin of tRFs: if they belong to the “tRNA-space” or to the “non-tRNA-space.” Indeed, in *white-* control *Drosophila* ovaries we observe a fraction of 15–17 nt long tRFs matching to the non-tRNA space (Supplementary Figure 1B). This proportion increases in *Rpp30^18.2^* mutants (Supplementary Figure 1C). Interestingly, if we run the same analysis excluding smallest tRFs (15–16 nt) profiles are similar in control (*w-)* while 5′tRFs accumulation in *Rpp30* mutants is less dramatic (Supplementary Figure 1E). Another problem in determining the origin of tRFs is that several tRNAs from the same amino-acid share the same sequence at different parts of the molecule^1^ (see alignments). Thus, sometimes we cannot distinguish if 5′-tRFs, 3′-tRFs or i-tRFs derived from a single or several tRNA molecules.

To analyze the expression of tRFs and have an idea of tRNA type forming tRFs, we made a tRNA heatmap reflecting the expression levels of all tRFs (all types comprised) belonging to a given tRNA isotype (Supplementary Figure 2) by using two different reference genomes: the “unique tRNA mature CCA-edited” (Supplementary Figures 2A,B) and the “tRNA extended CCA-edited” (Supplementary Figure 2C). By using the collapsed “unique tRNA mature sequences,” tRFs-1 originating from tRNAs precursors cannot be studied, neither taRFs or spanner tRFs.

In *white*^–^ control ovaries, among the most abundant tRFs originating from mature tRNA sequences we could observe: tRNA:Phe-GAA, tRNA:Val-AAC or TAC, tRNA:Lys-CTT, tRNA-Gly-GCC, tRNA:Pro-AGG or CGG, tRNA:His-GTG, and tRNA:Glu-CTC (Supplementary Figure 2A). If we study all types of tRFs by using the “tRNA extended CCA-edited” sequences we observe that tRFs from tRNA:Val-TAC or AAC were the most abundant, followed by tRFs mapping tRNA:Glu-CTC, several tRNA:Phe-GAA, and tRNA:Pro-CGG or AGG. tRFs corresponding to mature tRNA:Val-CAC, tRNA:Ala-TGC, tRNA:Lys-TTT or tRNA:Gln-CTG were also abundant. It is important to note that, as mentioned, tRNA modifications can induce sequencing bias allowing preferential sequencing to certain tRNA and tRFs types over others, since some tRNA (and potentially also tRFs) modification patterns are isoacceptor specific. Thus, the biological meaning of this tRFs abundance pattern remains to be explored.

To describe in more detail the most relevant tRFs profiles corresponding to each individual tRNAs, we developed a multidimensional tRFs map which displays the name of the tRNA molecule, the read counts and the tRFs position along the tRNA molecule (Figure 2E). For example, in control ovaries, highly expressed tRFs from tRNA:Val-CAC-2-3 produce mostly 3′-tRFs (dark blue) and 5′-tRFs to a lesser extent (light blue). Moreover, tRNA:Gln-CTG-4-1, a tRNA which generates high amounts of tRFs in control ovaries, has a clear majority of tRFs-1 (green).

In conclusion, our analysis describes in detail the population of tRFs present in control *Drosophila* ovaries in a global manner (annotation, coverage, size distribution, logo and heatmap tools), as well as the specific tRFs profiles of each tRNA isotype (multidimensional tRFs maps). We find that tRFs-1 are highly present, followed by 3′-tRFs and 5′-tRFs.

### tRNA Processing Defects Lead to tRFs Accumulation

**FIGURE 3:**
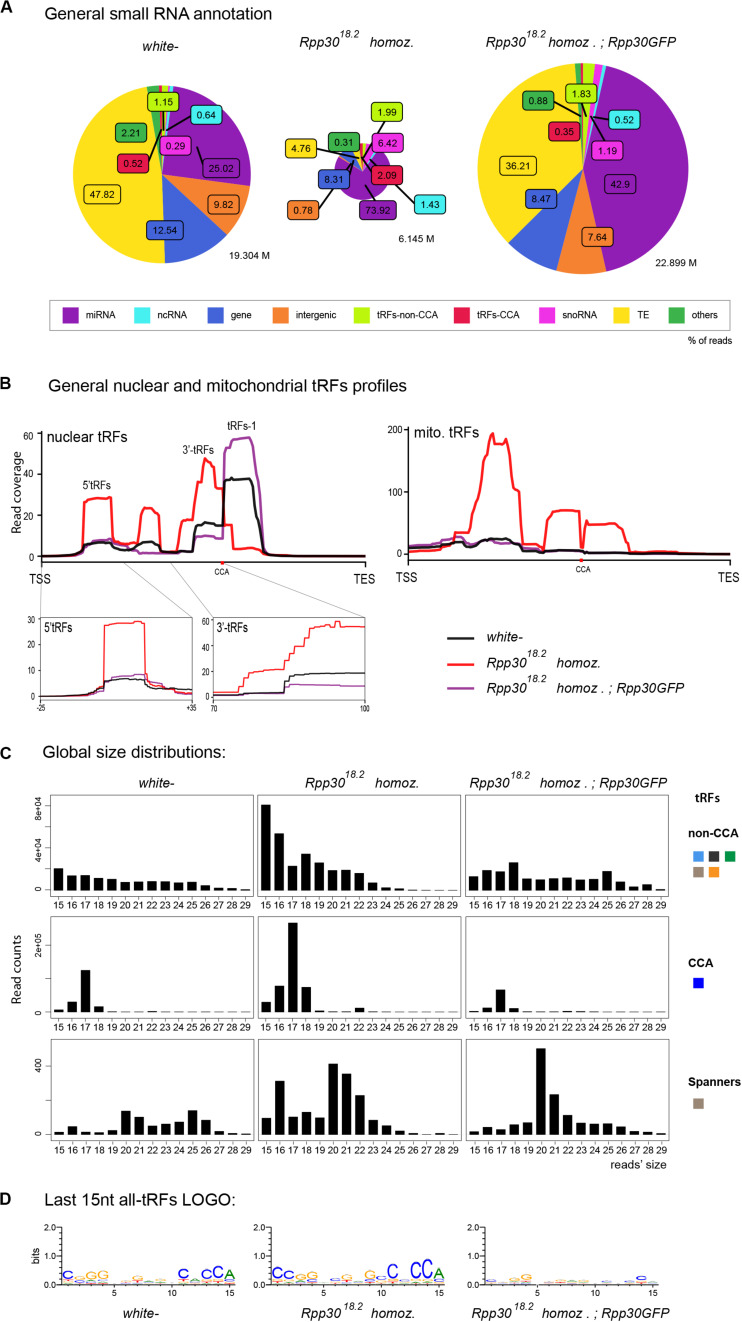
tRNA processing plays a role in nuclear and mitochondrial tRFs formation: **(A)** Small RNAs sequences (from 15–29 nt) were analyzed in different genotypes to distinguish categories with the help of an annotation cascade tool in the following order: miRNA, ncRNA, intergenic, genes, TE (piRNA, siRNA), snoRNAs, tRFs-non-CCA or tRFs-CCA. The percentage of reads for each genotype is shown in pie-charts, which size reflects the depth of each bank (M = Millions of reads). **(B)** Nuclear and mitochondrial tRFs coverages were analyzed in *white-* control and *Rpp30* mutant ovaries using scaling factors (see section “MATERIALS AND METHODS”). Different tRFs are shown along the coverage profile from the beginning of the pre-tRNA molecule (TSS transcription start site) to the end of the extended edited reference genome (TES, transcription extended site). CCA edition point is shown with a red dot. 5′-tRFs and 3′-tRFs regions are zoomed in, for a better comparison between the genotypes. **(C)** General size distribution (15–29 nt) of normalized read counts corresponding to the different categories of tRFs in *white-* control and mutant ovaries. Color-codes on the right are the same as in Figure 1B for tRFs categories. **(D)** Logo for the last 15 nt tRFs sequences of *white-* control and mutant ovaries (all categories included, issued from *fasta* files containing all tRFs sequences).

We recently discovered that *Drosophila Rpp30* mutations lead to tRNA processing and early oogenesis arrest, producing atrophied small ovaries full of early arrested stages (Molla-Herman et al., 2015). As control, we chose *white-* young (freshly hatched) ovaries described above, since they are full of early stages. Besides, we observed that *Rpp30* mutants have a defect in piRNA production. In accordance, cascade annotations showed that *Rpp30*^18.2^ homozygous ovaries have highly decreased TE-matching sequences compared to *white*^–^ (Figure 3A), which is rescued in *Rpp30*^18.^*^2;^ ubiRpp30GFP* ovaries, showing the specificity of the phenotype. Intriguingly, we observed a substantial increase of small RNAs derived from snoRNA (pink, 6.42% in the ovaries from *Rpp30*^18.2^ homozygous flies compared to 0.29%, observed in *white*^–^ controls). Moreover, we found that in *Rpp30*^18.2^ homozygous ovaries, both non-CCA and CCA-tRFs were present in equal quantities (1.99 *vs* 2.09%), whereas in control ovaries non-CCA-tRFs were more represented than CCA-tRFs (1.15% *vs* 0.52%). This suggests an increase of CCA-tRFs and/or a decrease of some non-CCA-tRFs in *Rpp30*^18.2^ homozygous mutants.

Nuclear tRFs coverage (Figure 3B, left panel) showed that in *Rpp30*^18.2^ homozygous ovaries, there is a substantial increase of 5′-tRFs, i-tRFs and 3′-tRFs), and a drastic decrease of tRFs-1, when compared to control. Importantly, rescued *Rpp30*^18.^*^2;^ Rpp30GFP* ovaries (purple line) showed a similar profile to *white*^–^, demonstrating that Rpp30 overexpression is able to recover tRFs formation in *Rpp30*^18.2^ homozygous mutants. In parallel, mitochondrial-tRFs coverages (Figure 3B, right) showed that *Rpp30*^18.^*^2^* homozygous individuals have a high accumulation of different tRFs types in their ovaries.

Next, global size distribution (Figure 3C) indicated that tRFs accumulate in *Rpp30*^18.2^ homozygous ovaries compared to *white*^–^. Indeed, non-CCA-tRFs range from 15 to 22 nt whereas 3′-tRFs are on average 17 nt long in mutants (Figures 3C,D). Finally, spanner-tRFs, which are a very minor population in *Drosophila white-* ovaries, are heterogeneous in size and do not show important changes in mutants when compared to control (Figure 3C, lower panels).

In conclusion, our analysis shows that tRNA processing defects alter tRFs biogenesis and/or stability in *Rpp30* mutants: increase of (5′-tRFs, i-tRFs and 3′tRFs), and tRFs-1 decrease. Since there are more than 300 tRNAs genes in *Drosophila*, we wondered if these defects were due to tRFs originating from a particular tRNA type.

### tRFs Expression Levels Are Altered in *Rpp30* Mutants

As mentioned, tRFs heatmaps showed that *white*^–^ control ovaries have abundant tRFs derived from tRNA-Val, Glu, Phe, Pro, Ala, Lys, Gln (Supplementary Figure 2). Importantly, the general heatmap profile is highly changed in *Rpp30*^18.2^ homozygous but is partially rescued in *Rpp30*^18.^*^2;^ ubiRpp30GFP* (Supplementary Figure 2). To easily detect the most drastic changes in tRFs populations we calculated the ratio of tRF-counts between *Rpp30*^18.2^ homozygous and *white-* ovaries (Supplementary Figure 2B). For example, tRFs derived from tRNA:Val-AAC-2-1 are highly decreased in *Rpp30*^18.2^ homozygous ovaries compared to *white*-, with a ratio of 0.05 (Supplementary Figure 2B).

**FIGURE 4:**
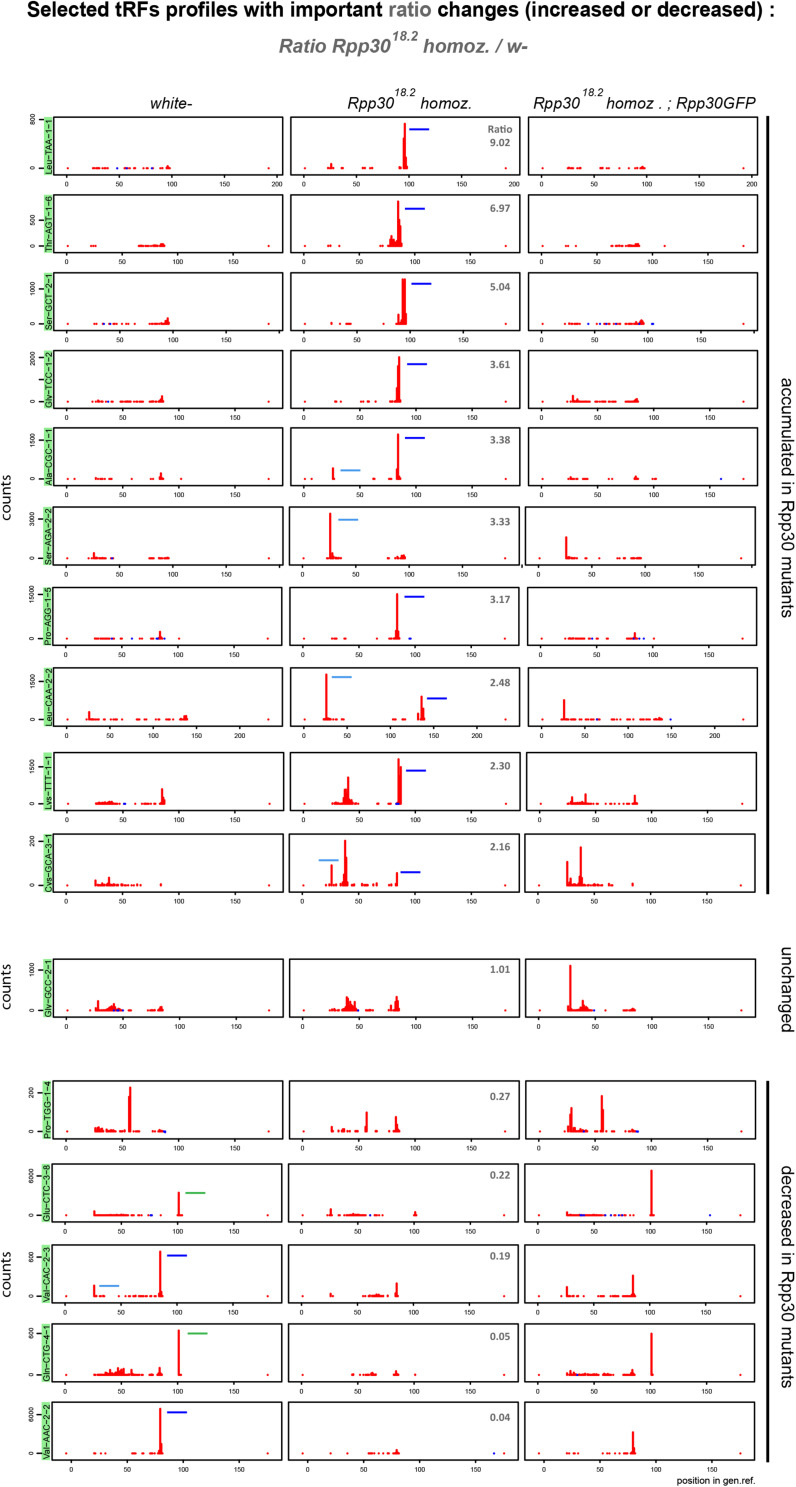
Rpp30 mutation leads to an increase of 5′-tRFs, an increase of 3′-tRFs and a decrease of tRFs-1. 16 tRFs readmap profiles as examples of the most increased or decreased tRFs from the ratio *Rpp30*^18.^*^2^ homoz./white-* (see in Supplementary Figure 2B) are shown for the different genotypes, using normalizing factors (see section “MATERIALS AND METHODS”). Since pre-tRNAs sequences are included in the tRNA reference genome, 5′-tRFs start at position 25 nt instead of position 0 nt. 3′-tRFs are located around the position 80 nt and tRFs-1 are located around position 100 nt (positions can vary depending on tRNA lengths and the presence of intron). Peaks determine the beginning of the reads sequences. tRFs are schematized in *white-* and *Rpp30*^18.2^ homozygous for better comparison: 5′-tRFs in light blue, 3′-tRFs in dark blue and tRFs-1 in green. Ratio’s values above 1 (upper pannels): tRFs increased in *Rpp30*^18.^*^2^* mutants. Ratio’s values below 1 (lower panels): tRFs decreased in *Rpp30*^18.^*^2^* mutants.

From this ratio data, we selected tRNA profiles in which tRFs were increased, decreased or unchanged in mutants when compared to *white-* (Figure 4). For example, in *Rpp30*^18.2^ mutants: tRNA:Leu−TAA−1−1, tRNA:Thr−AGT−1−6, tRNA:Ser−GCT−2−1, tRNA:Gly−TCC−1−2 and tRNA:Pro−AGG−1−5 show an increase of 3′-tRFs. In addition, tRNA:Ala−CGC−1−1 accumulates 3′−tRFs and 5′−tRFs. tRNA:Ser−AGA−2−2 shows a drastic increase in only 5′−tRFs. Indeed, all tRNA:Ser−AGA/CGA (12 different tRNAs) behave similarly. tRNA:Leu−CAA−2−2 has an important increase in 5′−tRFs as all tRNA:Leu−CAA. It should be noted that Leu-CAA group have an intron of 40–44 nt, that is why 3′-tRFs are located offset in tRFs maps. Next, tRNA-Gly-GCC-2-1 is similar in *white-* and *Rpp30*^18.2^ mutants. Finally, several tRFs types decreased in *Rpp30*^18.2^ mutants: tRFs-1 generated from tRNA:Glu-CTC-3-8 and tRNA:Gln-CTG-4-1; 5′-tRFs generated from tRNA:Val-CAC-2-3; 3′-tRFs generated from tRNA:Val-CAC-2-2 and 2-3. We also compared tRFs profiles by selecting tRNAs having the mostly expressed tRFs (up to heatmaps) in *white-* and we compared them to mutants (Supplementary Figure 3).

Overall, we find that in *Drosophila* ovaries, tRFs originate from diverse isotypes of tRNAs and show heterogeneous profiles. In general, as shown in Figure 3B, we find that tRFs-1 are decreased in *Rpp30* mutants, whereas tRFs originating from mature tRNA are accumulated. tRNA processing by RNase P is the first step of tRNA biogenesis following transcription. We thus wondered whether other downstream events could also affect tRFs biogenesis or stability, such as tRNA post-transcriptional modifications of tRNA molecules.

### tRNA 2′-O-Methylation Defects Lead to a Decrease of tRFs-1 and an Increase of 3′-tRFs

**FIGURE 5:**
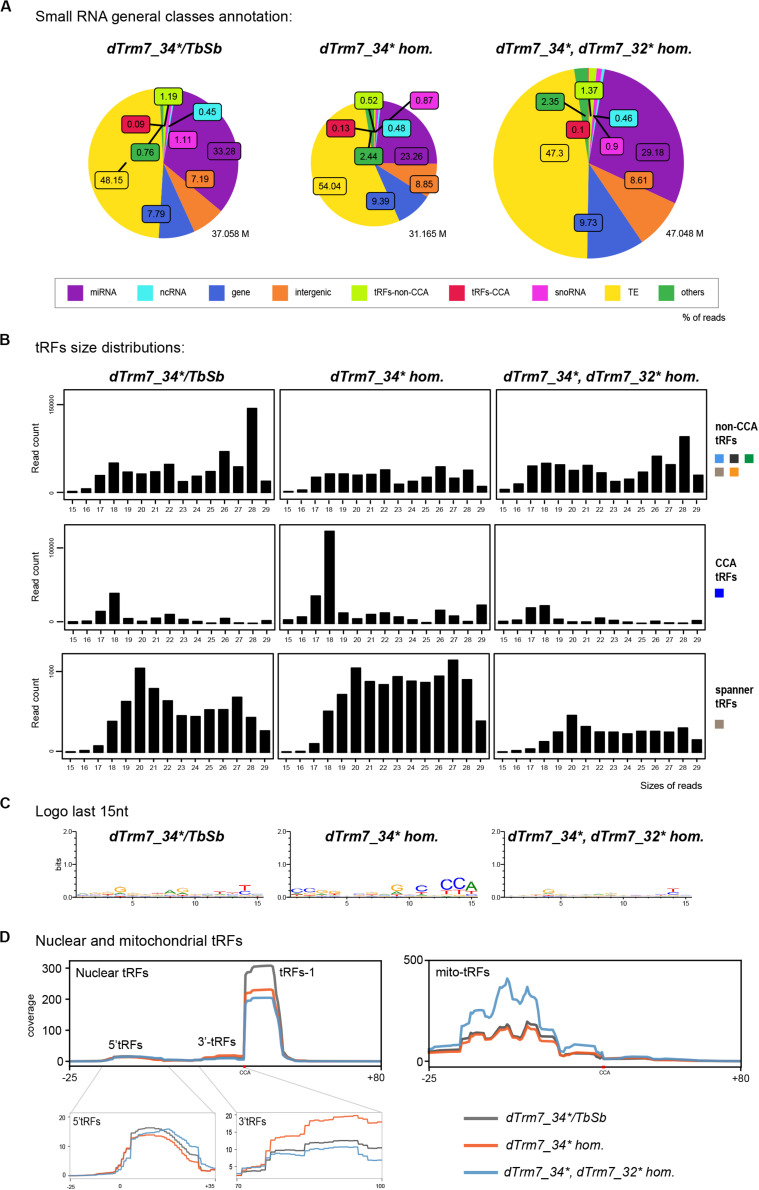
tRNAs methylation defects alter nuclear and mitochondrial tRFs formation: **(A)** Small RNAs sequences from 15–29 nt were analyzed in different genotypes to distinguish different categories with the help of an annotation cascade tool in the following order: miRNA, ncRNA, intergenic, genes, TE (piRNA, siRNA), snoRNAs, tRFs-non-CCA or tRFs-CCA. The percentages of reads from *dTrm7_34^∗^* heterozygous, *dTrm7_34^∗^* homozygous and *dTrm7_34^∗^, dTrm7_32^∗^* double mutant ovaries are shown in pie-charts. The pie-charts size reflects the depth of the bank (M = Millions of reads). **(B)** General size distribution (15–29 nt) of normalized read counts corresponding to the different categories of tRFs in different genotypes using scaling factors (see section “MATERIALS AND METHODS”). Color-codes for the tRFs categories on the right are described in Figure 1B. **(C)** Logo for the last 15 nt tRFs sequences of control and mutant ovaries (all categories included, issued from *fasta* files). **(D)** Nuclear and mitochondrial tRFs coverages were analyzed in different genotypes using scaling factors (see section “MATERIALS AND METHODS”). Different types of tRFs are shown along the coverage profile from the beginning of pre-tRNA (TSS transcription start site) to the end of the extended edited reference genome (TES, transcription extended site). CCA edition point is shown with a red dot. 5′-tRFs and 3′-tRFs regions are zoomed in for better comparison between the genotypes.

Mutations of tRNAs 2′-O-methyltransferases (Nm MTases) *dTrm7_34* and *dTrm7_32* lead to *Drosophila* life span reduction, small RNA pathways dysfunction, increased sensitivity to RNA virus infections and tRFs-Phe accumulation (Angelova et al., 2020). In our cascade annotation analysis (non-normalized), non-CCA-tRFs decrease in *dTrm7_34^∗^* homozygous mutants when compared to control (Figure 5A, light green, 1.19% versus 0.52%), whereas tRFs-CCA slightly increase (Figure 5A, red, 0.09% versus 0.13%). Surprisingly, double mutants *dTrm7_34^∗^, dTrm7_32^∗^* show profiles similar to control. By using normalization factors, the analysis of tRFs size distribution and of a Logo sequence revealed that 3′-tRFs of 18 nt increase in *dTrm7_34^∗^* homozygous mutants when compared to control (Figure 5B, C), which was rescued in double mutants. Finally, lowly expressed spanner tRFs were similar in *dTrm7_34^∗^* heterozygous and homozygous mutants when compared to control and slightly lower in double mutants (Figure 5B).

To obtain an overview of which tRFs classes were globally altered in Nm MTases mutants, we aligned all-tRFs together along a canonical nuclear or mitochondrial tRNA precursors. In heterozygous control ovaries (Figure 5D, gray), there is a majority of nuclear tRFs-1, similarly to control *white-* ovaries (Figure 2B). Of note, the size of heterozygous ovaries is bigger than *white-* ovaries, since they have early and older stages. Interestingly, *dTrm7_34^∗^* homozygous mutants and double mutants *dTrm7_34^∗^, dTrm7_32^∗^* showed a decrease of tRFs-1 when compared to heterozygous control (Figure 5D, orange and blue), suggesting that these Nm MTases are involved in tRFs biogenesis and/or stability.

Since tRFs-1 reads signal is very high and the signal of 5′-tRFs and 3′-tRFs is lower, it was difficult to identify major changes in tRFs originating from mature tRNAs. By zooming into these regions, we observed that 5′-tRFs slightly decrease in *dTrm7_34^∗^ homozygous* mutants (Figure 5D, left panel, orange), whereas 3′-tRFs increase (Figure 5D, right panel, orange). In this analysis double mutants again show similar profiles to control, suggesting that *dTrm7_32* mutation somehow rescues *dTrm7_34* defects on 3′-tRFs accumulation. Interestingly, we recently reported that longer 5′-tRF-Phe (∼35 nt) were significantly increased in different combinations of *dTrm7_34* mutant alleles (Angelova et al., 2020).

Moreover, we observed mitochondrial tRFs in heterozygous ovaries similar to *white-* flies (Figures 2B, 5D, right panel, gray line), derived mostly from the first half of the molecule. Homozygous mutant for *dTrm7_34^∗^* ovaries are similar to heterozygous mutants, whereas double mutants *dTrm7_34^∗^, dTrm7_32^∗^* show a global increase of mito-tRFs, suggesting that *dTrm7_32*, and not *dTrm7_34*, may be involved in mitochondrial-tRFs biogenesis and/or stability.

In summary, we have observed that defects of tRNA 2′-O-methylation affect tRFs populations in *Drosophila* ovaries. *dTrm7_34* and *dTrm7_32* mutations lead to a decrease of tRFs-1 and *dTrm7_34* mutation leads to an accumulation of 3′-tRFs and a slight decrease of 5′-tRFs.

### tRNA Methylation Mutations Affect tRFs Derived From Different Isotypes of tRNAs

tRNA expression heatmaps using “extended tRNA CCA-edited reference genome” allowing the analysis of all types of tRFs showed that the most expressed tRFs in ovaries from heterozygous *dTrm7_34^∗^* mutants were those corresponding to tRNAs Glu-CTC, Pro-CGG and AGG, Val-TAC, Cys-GCA, Lys-TTT, Gly-TCC, Ala-CGC, His-GTG, Ser-GCT (Supplementary Figure 4A), similarly to *white-* control ovaries (Supplementary Figure 2C). In the ovaries from *dTrm7_34^∗^* homozygous mutants, we observed a decrease of the tRFs derived from Glu-CTC, Cys-GCA, Lys-TTT or Gly-TCC, whereas tRFs derived from Ser-GCT were increased when compared to control. These changes have been quantified by calculating the ratio between homozygous and heterozygous *dTrm7_34^∗^* read counts (Supplementary Figure 4B).

**FIGURE 6:**
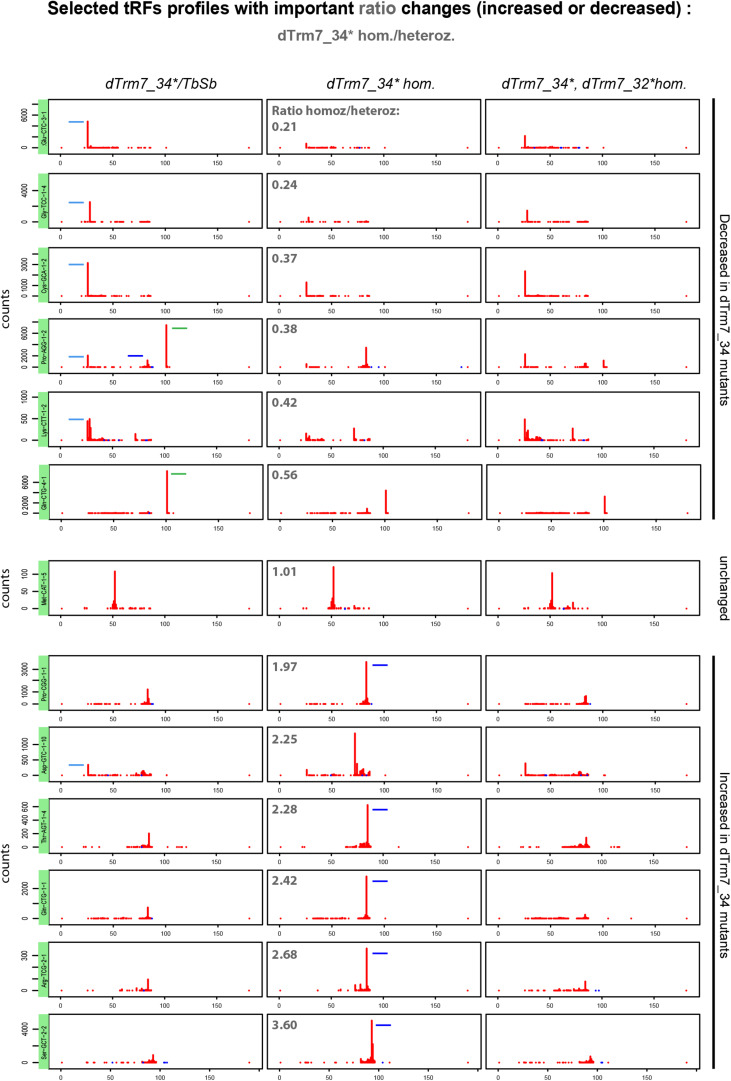
tRFs expression is altered in tRNA methylation mutants: 13 tRFs readmap profiles as examples of the most increased or decreased tRFs from the ratio *dTrm7_34^∗^ homozygous/heterozygous* (see in Supplementary Figure 4B) are shown for the different genotypes, using normalizing factors (see section “MATERIALS AND METHODS”). Since pre-tRNAs sequences are included in the tRNA reference genome, 5′-tRFs start at position 25 nt instead of position 0 nt. 3′-tRFs are located around the position 80 nt and tRFs-1 are located around position 100 nt, depending on tRNA lengths and the presence of intron. Peaks determine the beginning of the reads sequences. tRFs are schematized in *dTrm7_34^∗^* homozygous and heterozygous mutants for better comparison: 5′-tRFs in light blue, 3′-tRFs in dark blue and tRFs-1 in green. Ratio’s values above 1 (lower panels): tRFs increased in *dTrm7_34^∗^ homozygous* mutants. Ratio’s values below 1 (upper panels): tRFs decreased in *dTrm7_34^∗^ homozygous* mutants.

Considering the read counts ratio change between homozygous and heterozygous *dTrm7_34^∗^* ovaries (Figure 6, upper panels), we observe that 5′-tRFs are strongly decreased for several tRNAs, such as Glu-CTC, Gly-TCC, Cys-GCA. This effect is partially rescued in double mutants. In addition, we observe that tRFs-1 from tRNAs Gln-CTG-4-1 and Pro-AGG-2-1 are strongly reduced in the ovaries from both homozygous *dTrm7_34^∗^ and dTrm7_32^∗^, dTrm7_34^∗^* compared to the control (Figure 6, upper panels). We detect no change in tRNA:Met-CAT-1-5 tRFs between control and mutants, where the observed tRFs population matches the anticodon region (Figure 6, middle panel). On the contrary, we clearly see an increase of 3′-tRFs in *dTrm7_34^∗^* homozygous mutants for several tRNAs: Pro-CGG-1-1, Thr-AGT-1-4, Gln-CTG-1-1, Arg-TCG-2-1, Ser-GCT-2-2 (Figure 6, lower panels). Surprisingly, those defects are rescued in double mutants *dTrm7_32^∗^, dTrm7_34^∗^*. In addition, we find similar results analyzing profiles corresponding to highly expressed tRFs in heterozygous *dTrm7_34^∗^* ovaries for 5′-tRFs and tRFs-1 (Supplementary Figure 5). However, increase of 3′-tRFs are difficult to observe, indicating that the increased in *dTrm7_34^∗^* homozygous ovaries 3′-tRFs are not highly present in heterozygous ovaries.

We recently showed that dTrm7_34 and dTrm7_32 methylate tRNA-Leu, Trp, Phe (conserved targets in yeast and humans), as well as that dTrm7_32 methylates tRNA-Glu and Gln in *Drosophila* (Angelova et al., 2020). Indeed, tRFs derived from these specific tRNAs show different profiles between mutants and control conditions (Supplementary Figure 6). First, tRNA-Leu tRFs have different profiles regarding their anticodon sequence. Some 5′-tRFs in control ovaries are decreased in *dTrm7_34^∗^* mutants and remain decreased or are rescued in double mutants. tRFs-1 decrease in *dTrm7_34^∗^* homozygous mutants, whereas 3′-tRFs increase. Thus, tRNA-Leu tRFs follow the general tendency observed in Figure 5D. In contrast, tRNA-Trp- and tRNA-Phe-derived 3′-tRFs increase in *dTrm7_34^∗^* homozygous mutants, while double mutants *dTrm7_34^∗^, dTrm7_32^∗^* lose 3′-tRFs.

**FIGURE 7:**
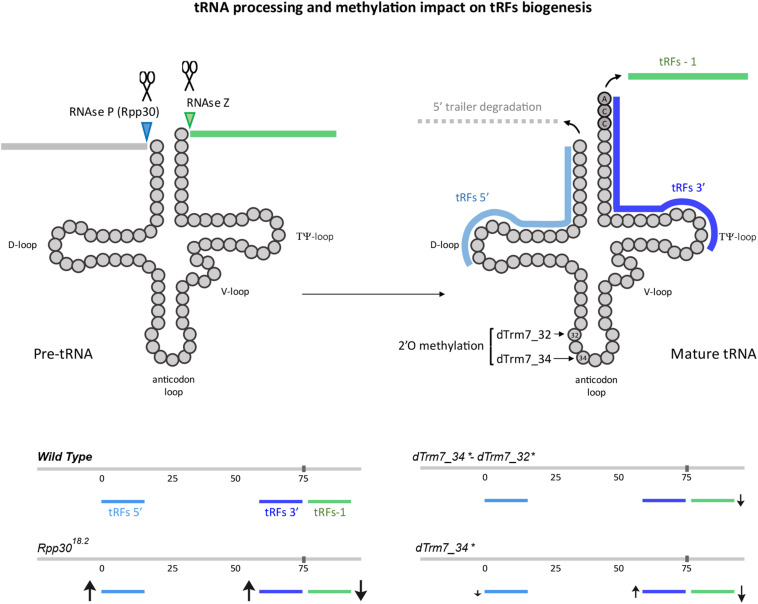
tRNA processing and methylation defects impact on tRFs biogenesis: The main steps of tRNA processing are depicted. Cleavage sites for ribozymes RNase P and Z are indicated on a pre-tRNA molecule. Cleavage of the 3′ trailer forms tRFs-1 (green). Upon cleavage of the leader and trailer sequences and CCA addition (dark gray), yielding mature tRNAs, they can be cleaved at the D-loop, forming the tRFs 5′ (light blue) and at the T-loop, forming 3′-tRFs. 2′O-methylation sites for dTrm7_32 and dTrm7_34 are shown at the anticodon loop. Increase or decrease of different tRFs populations in mutants for tRNA processing or tRNA methylation are schematized with arrows of different sizes (↑, increased, ↓, decreased).

Overall, tRNA Nm methylation defects in the anticodon loop have a global impact on tRNA fragmentation, though to a lesser extent than tRNA processing defects (Figure 7). Indeed, tRFs-1 show a decrease in *dTrm7_34^∗^* homozygous mutants and 3′-tRFs are increased, whereas 5′-tRFs are slightly decreased. Intriguingly, double mutants *dTrm7_34^∗^, dTrm7_32^∗^* have profiles similar to control, indicating that for at least some of the observed differentially expressed tRFs increased in *dTrm7_34^∗^* homozygotes, dTrm7_32-dependent Nm modification might have an effect on their biogenesis and/or stability. Finally, tRFs longer than 30 nt can’t be properly detected in the analyzed libraries (size selection of 15–29 nt), so our analysis does not include the 35 nt long tRNA-Phe-derived 5′-tRFs characterized previously in *dTrm7_34^∗^* mutants (Angelova et al., 2020).

### tRNA Processing and Methylation Avoid snoRNA Fragmentation

The increase of small RNAs derived from snoRNAs observed in *Rpp30* mutants (Figure 3A) led us to study this population in more detail. In *Drosophila*, snoRNAs > 120 nt are box H/ACA and play a role in pseudouridylation whereas snoRNAs < 120 nt are box C/D snoRNA and play a role in 2′-O-methylation (Huang, 2005; Angrisani et al., 2015; Falaleeva et al., 2017). Since RNase P has been shown to participate in snoRNAs maturation in some species (Coughlin et al., 2008) and since snoRNAs molecules can be cleaved to form snoRNA fragments (snoRFs) by enzymes that remains to be elucidated (Falaleeva and Stamm, 2012; Światowy and Jagodzińśki, 2018), we studied a potential role of RNase P in snoRFs biogenesis (Supplementary Figure 7).

snoRFs size distributions shows that snoRFs are highly increased in *Rpp30*^18.2^ homozygous mutants, with snoRFs mostly ranging between 15 and 23 nt (Supplementary Figure 7A). Since there are two main snoRNA populations (box H/ACA and C/D Supplementary Figure 4B), we analyzed them all together and separately to observe snoRFs coverages. Indeed, total snoRFs coverage shows that in control flies (*white-* and rescued *Rpp30^18.2^; Rpp30GFP*) there is almost no snoRFs formation (Supplementary Figure 7C, upper panel, black and purple lines). However, snoRFs are highly increased in *Rpp30*^18.2^ homozygous mutants (red line), mostly in 3′ of snoRNA molecules. Indeed, there is a strong accumulation of box C/D snoRFs mostly at 3′ of the snoRNA molecule, and an increase of box H/ACA 5′ and 3′ snoRFs in ovaries from *Rpp30* mutants compared to controls. The sequence specificities for box C/D or H/ACA can be observed by analyzing the Logo (Supplementary Figure 7D).

In methylation mutants, ovaries from heterozygous and homozygous *dTrm7_34^∗^* mutants show similar profiles, with snoRFs mostly ranging from 21-28 nt (Supplementary Figure 7A). Indeed, in comparison to *white-* where almost no snoRFs are detected (Supplementary Figure 7C), we observe that snoRFs accumulate in tRNA methylation mutant genetic backgrounds (heterozygous, homozygous and double mutants), mostly at the 3′ part of snoRNA molecules (Supplementary Figures 7A, C). These results suggest that dTrm7_34 and dTrm7_32 function(s) can be important in avoiding snoRFs fragmentation.

## Discussion

Our study presents an easy to share user friendly bioinformatic workflow for tRFs population analysis and its use on Illumina-generated small RNA libraries. As proof of principle we used libraries of control and mutant *Drosophila* for two key events of tRNA biology: tRNA processing and tRNA Nm methylation at the anticodon loop. We provide a new genome reference, comprising sequences upstream and downstream of mature tRNA genome sequences and bioinformatically added CCA tags that allow analysis of 3′-tRFs and 5′-tRFs, i-tRFs, tRFs-1, taRFs and spanners (Figure 1A).

Using mutant flies for the RNAse P subunit (*Rpp30*^18.2^) we observed an important decrease in tRFs-1 (Figure 7). tRFs-1 are generated by RNase Z-mediated cleavage of pre-tRNAs. Interestingly, it has been described in *Drosophila* and other species that RNase P cleaves the 5′ trailer before RNase Z cleaves the 3′ trailer (Dubrovsky et al., 2004; Xie et al., 2013). In this way, an upstream defect on 5′ cleavage due to *Rpp30* mutation could affect RNase Z cleavage, thereby explaining why tRFs-1 decrease in *Rpp30* mutants. Moreover, *Rpp30*^18.2^ mutants show an accumulation of 5′-tRFs. It is possible that a lack of 5′ leader cleavage affects tRNA secondary structure, promoting cleavage in the D-loop to form 5′-tRFs by Dicer or other endonucleases as already shown in mammals (Li et al., 2018). Finally, 3′-tRFs also increase in *Rpp30*^18.2^ mutants. CCA is known to be added on mature tRNA, which suggests that Rpp30 mutation somehow affects tRNA cleavage after the CCA tRNA editing. Since 3′-tRFs are involved in TEs silencing control, increasing this tRFs population by promoting tRNA cleavage at the T-loop can be a way to control TEs when the main piRNA pathway is compromised. This observation is consistent with previous reports of tRFs functioning as a versatile and adaptive source for genome integrity protection (Martinez et al., 2017; Schorn et al., 2017). Also, it is important to mention that *Rpp30* mutants accumulate short tRFs (15–17 nt) which origin is difficult to know: tRNA-space versus non-tRNA space. Indeed, when 15–16 nt are excluded from the analysis, while control profiles do not change, the tRFs increase is less dramatic (Supplementary Figure 1E), suggesting that they could partly correspond to non-tRNA space. For example, they could originate from TEs overexpression and fragmentation, but this remains to be elucidated.

Besides tRFs, we observed that snoRNAs fragments (snoRFs) accumulate in *Rpp30* homozygous mutant ovaries. In this sense, it has been shown that snoRNAs can be a target of RNase P in some species during snoRNA maturation (Coughlin et al., 2008; Marvin et al., 2011). We know now that snoRNAs molecules can be cleaved into snoRNA fragments (snoRFs) but the enzyme(s) responsible for their cleavage remain(s) poorly characterized (Światowy and Jagodzińśki, 2018). snoRFs are aberrantly present in several pathologies such as cancer and neurodegenerative diseases (Falaleeva and Stamm, 2012; Patterson et al., 2017; Romano et al., 2017; Światowy and Jagodzińśki, 2018). It is thus possible that RNase P limits snoRNA fragmentation to preserve homeostasis by an uncharacterized mechanism. Interestingly, mice mutant for RNase Z (the other major tRNA processing enzyme) showed an increase in snoRNAs expression. This phenomenon was proposed to compensate translation defects produced by the lack of correct 3′ tRNA processing (Siira et al., 2018). However, a role of RNase Z in snoRFs formation has not been described.

As introduced previously, in *Drosophila* some methylation marks protect tRNAs from cleavage and aberrant tRFs populations accumulate in mutants for different methyltransferases, such as Dnmt2 (catalyzes m^5^C methylation) and dTrm7_32 and dTrm7_34 (catalyze 2′-O-methylation) (Schaefer et al., 2010; Durdevic et al., 2013b; Genenncher et al., 2018; Angelova et al., 2020). In addition, It has recently been shown in mice that loss of NSUN2 altered tRFs profiles in response to stress, impairing protein synthesis (Gkatza et al., 2019). Our analysis of the tRFs populations in ovaries mutant for dTrm7_34 and dTrm7_32, two Nm MTases of the anticodon loop of some tRNAs, showed that *dtrm7_34* mutants have different tRFs profiles when compared to *Rpp30*^18.2^ mutants (Figure 7): tRFs-1 are decreased compared to control, 5′-tRFs are slightly decreased, whereas 3′-tRFs are increased. dTrm7_34 has been shown to methylate tRNAs at the wobble position 34 of the anticodon region and its mutation leads to an accumulation of tRNA halves fragments (around 35 nt length) (Angelova et al., 2020). Thus, an accumulation of longer tRFs could impede a cleavage in the D-loop, explaining a decrease in 5′-tRFs. However, in this study we cannot detect tRNA halves since our datasets contain RNAs of 15–29 nt only. Moreover, 3′-tRFs increase in *dTrm7_34^∗^* homozygous mutants, suggesting that tRNA Nm methylation at position 34 somehow limits T-loop cleavage. The other anticodon Nm methyltransferase, dTrm7_32, has been shown to methylate position 32 of its substrate tRNAs (Angelova et al., 2020). Interestingly, double mutants *dTrm7_34^∗^, dTrm7_32^∗^* show a different tRFs profile when compared to *dTrm7_34^∗^* single mutant. This result suggests that a lack of methylation in the anticodon loop region can somehow favorize the production and/or stabilize some tRFs, as proposed recently for tRNA halves in Nm mutants (Angelova et al., 2020).

Finally, our study detected an increase of mitochondrial derived tRFs in *Rpp30*^18.2^ mutants, as well as in double mutants *dTrm7_34^∗^, dTrm7_32^∗^* when compared to control, whereas control conditions show very low levels of mito-tRFs. This observation indicates that tRNA processing and tRNA Nm methylation pathways of the anticodon loop limit aberrant fragmentation of mitochondrial tRNAs. Mito-tRNAs are polycistronic sequences cleaved by conserved mitochondrial RNase P and Z complexes in several species (Jarrous and Gopalan, 2010; Rossmanith, 2012). Intriguingly, a recent study reported an interplay between RNase P complex and mito-tRNA methylation enzymes in human cells. Indeed, mito-RNAse P was shown to recognize, cleave and methylate some mitochondrial tRNAs *in vitro*, and its activity was enhanced by interaction with a tRNA methylation cofactor (Karasik et al., 2019). Mito-RNAse P and Z dysfunctions have also been linked to several human mitochondrial diseases, as myopathies and neurodevelopmental disorders (Barchiesi and Vascotto, 2019; Saoura et al., 2019). A description of mitochondrial tRFs biogenesis could thus help to better understand the molecular mechanisms underlying these pathologies. In line with neurodegenerative diseases implication, tRFs have been shown to be present in the brain of different species, and their populations were shown to vary during aging in *Drosophila* (Karaiskos et al., 2015; Karaiskos and Grigoriev, 2016; Angelova et al., 2018).

High throughput Illumina sequencing of small RNA libraries could introduce biases in tRFs detection, since tRNAs are highly modified molecules and very few techniques are able to properly describe these modifications in a transcriptome-wide way, such as ARM-seq or Circ-RNA-seq tRNA (Cozen et al., 2015; Zhang et al., 2015). For example, in *white-* control ovaries, tRFs-1 are the most highly present, followed by 3′-tRFs and 5′-tRFs. This tRF distribution in the sample could be due to the method of library preparation or sequencing, since with standard small RNA-Seq protocols, tRFs-1 could be preferentially sequenced as they are poorly modified post-transcriptionally. In addition, since the reverse transcription occurs from the 3′-end of the tRNA sequence, because of tRNA modifications libraries could be biased toward detection of reads mapping to the 3′-end of tRNA sequences (Torres et al., 2019). However, some studies have reported that tRNA modifications only have a limited impact on data mining when studying tRFs in The Cancer Genome Atlas (Telonis et al., 2019). Importantly, a huge number of datasets are already available with valuable information to extract. By analyzing different mutants from distinct pathways we should be able to increase our knowledge on tRFs biogenesis and/or stability, as well as on the potential interactions between the diverse mechanisms impacting tRFs biology. For example, it has been recently shown that snoRNAs can 2′-O-methylate tRNA-CAT at position 34 in mammalian cells, similarly to dTrm7_34 (Vitali and Kiss, 2019; Angelova et al., 2020). Conversely, tRNA methylation could have an impact on snoRNAs biogenesis, as observed in this study. Thus, our new workflow can help to analyze past, present and future small RNA sequences obtained by different means. It will be interesting to obtain a tRF cartography in different tissues, organs and species; to determine tRFs targets and biogenesis factors; as well as to elucidate tRFs functions in gene expression regulation. It will also be interesting to compare datasets obtained from classical Illumina sequencing with other techniques such as ARM-seq, which provides a read out of some modifications and may reveal additional tRFs populations. Our study thus has the potential to participate in the discovery of novel nuclear or mito-tRFs that could help advance the understanding of the etiology of a wide range of human pathologies.

## Data Availability Statement

The datasets generated for this study can be found in the European Nucleotide Archive (ENA) http://www.ebi.ac.uk/ena, accession numbers: PRJEB10569 (Rpp30 mutants), PRJEB35301 and PRJEB35713 (Nm mutants).

## Author Contributions

AM-H and MA performed experiments. AM-H and CA designed and performed bioinformatic data analyses. AM-H wrote the manuscript. J-RH participated in data analysis and manuscript writing. MA, CA, and CC participated in manuscript writing. MG did the comparative table of tRFs analysis methods. All authors contributed to the article and approved the submitted version.

## Conflict of Interest

The authors declare that the research was conducted in the absence of any commercial or financial relationships that could be construed as a potential conflict of interest.

## Footnotes

1 http://gtrnadb.ucsc.edu/

2 http://gtrnadb.ucsc.edu/genomes/eukaryota/Dmela6/Dmela6-align.html

3 www.ebi.ac.uk/ena

4 www.ncbi.nlm.nih.gov/geo

5 https://mississippi.snv.jussieu.fr

6 http://www.ebi.ac.uk/ena

7 http://www.ensembl.org/biomart/martview

## Supplementary Material

Supplementary Figure 1 tRFs could originate from tRNA space and non-tRNA space: **(A)** Representation of the different genomic loci that can give rise to tRFs: tRNAs, tRNA-lookalikes, truncated tRNAs or repeated elements. **(B)** tRFs size distribution of reads matching “tRNA space” (gray) and “non-tRNA space” (blue). **(C)** Fold change of tRF reads matching the “tRNA-space” versus tRFs reads matching the “non-tRNA space.” **(D)** tRFs matching the “non-tRNA space” corresponding to tRFs-CCA (dark pink) and tRFs-non-CCA (light pink) in the represented genotypes. **(E)** comparison between Bam Coverages obtained with 15–29 nt (left, same as Figure 3B) and 17–29 nt (right) in different genotypes.

Supplementary Figure 2 tRFs expression is altered in Rpp30 mutants: **(A)** Unique mature-tRNA CCA-tagged sequences were used as a reference genome to identify tRNAs that are sources of tRFs. tRFs reads were counted in a hit table, normalized by DESeq normalization Geometrical method and used to generate a heatmap for the different genotypes (1-3) reflecting all nuclear and mitochondrial tRNA genes in *Drosophila*’s genome. The read counts were sorted from maximum to minimum values for the *white-* column (left) for a better comparison with the other genotypes. Expression levels are reflected with a color-code going from blue (lowest levels), through white (middle levels), to red (highest levels) and ranging from 0 to 15 log2 counts. **(B)** Ratio of the read counts *Rpp30*^18.^*^2^* homozygous/*white-* presented in **(A)** Minimal values are in dark blue, middle values are in white, and maximal values are in red. **(C)** Heatmap generated as in **(A)**. by using “tRNA-extended CCA edited” sequences in order to analyze all types of tRFs: from precursor and mature tRNAs.

Supplementary Figure 3 Rpp30 mutations affects tRFs biogenesis: 11 tRFs readmap profiles representing the most expressed tRFs in *white-* (see Supplementary Figure 2C) are shown for the different genotypes. The readmaps were obtained using normalizing factors (see Methods). Since pre-tRNAs sequences are included in the tRNA reference genome, 5′-tRFs are located at position 25 nt instead of position 0 nt. 3′-tRFs are located around position 75 nt and tRFs-1 are located around position 100 nt, depending on the length of the tRNAs and the presence of intron. Peaks determine the beginning of the reads sequences. tRFs categories are schematized in *white-* and *Rpp30*^18.2^ homozygous for better comparison: 5′-tRFs in light blue, 3′-tRFs in dark blue and tRFs-1 in green.

Supplementary Figure 4 tRFs biogenesis is altered in tRNA methylation mutants: **(A)** tRFs were counted in a hit table using tRNA-extended CCA edited as a reference genome. The table was normalized by DESeq normalization Geometrical method and used to generate a heatmap for the different genotypes (1-3) reflecting all nuclear and mitochondrial tRNA genes in *Drosophila*’s genome. The read counts were sorted from maximum to minimum values for the *white-* column (left) for better comparison with the other genotypes. Expression levels are reflected with a color-code going from blue (lowest levels), through white (middle levels), to red (highest levels). **(B)** Read counts ratio *dTrm7_34^∗^ homoz./heteroz.* calculated from **(A)** Minimal values are in dark blue, middle values are in white, and maximal values are in red, ranging from 0 to 15 log2 counts.

Supplementary Figure 5 tRNA methylation defects alter tRFs populations: 12 tRFs normalized readmap profiles representing the most decreased or increased tRFs from the ratio *dTrm7_34^∗^* homozygous/heterozygous (see Supplementary Figure 4B) are shown for the different genotypes. Since pre-tRNAs sequences are included in the tRNA-reference, 5′-tRFs are located at position 25 nt instead of position 0 nt. 3′-tRFs are located around position 75 nt and tRFs-1 are located around position 100 nt, depending on the length of the tRNAs and the presence of an intron^∗^. Peaks determine the beginning of the reads sequences. The tRFs categories are schematized in *dTrm7_34^∗^/Tb,Sb* heterozygous for better comparison: 5′-tRFs in light blue, 3′-tRFs in dark blue and tRFs-1 in green. Ratio’s values above 1: tRFs increased in *dTrm7_34^∗^* homozygous mutants. Ratio’s values below 1: tRFs decreased in *dTrm7_34^∗^* homozygous mutants.

Supplementary Figure 6 Lack of dTrm7_32 and dTrm7_34 affects the abundance of tRFs, derived from their substrate tRNAs: 13 tRFs normalized readmap profiles as examples of tRNA substrates of dTrm7_34 and dTrm7_32 are shown for the different genotypes. Since pre-tRNAs sequences are included in the tRNA-reference, 5′-tRFs are located at position 25 nt instead of position 0 nt. 3′-tRFs are located around position 75 nt and tRFs-1 are located around position 100 nt, depending on the length of the tRNA and the presence of an intron. Peaks determine the beginning of the reads sequences. tRFs are schematized in *dTrm7_34^∗^/Tb,Sb* heterozygous and homozygous mutants for better comparison: 5′-tRFs in light blue, 3′-tRFs in dark blue and tRFs-1 in green.

Supplementary Figure 7 tRNA processing and tRNA methylation affects snoRNA fragments (snoRFs) profiles. **(A)** General size distribution (15–29 nt) of normalized snoRFs read counts is shown for the different genotypes of tRNA processing and tRNA methylation mutants. **(B)** Violin plot reflecting snoRNAs populations found in *Drosophila melanogaster* genome. snoRNAs of more than 120 nt belong to *box H/ACA* class whereas snoRNAs of less than 120 nt belong to *box C/D* class. **(C)** Shown are snoRFs coverage profiles for the indicated genotypes (scaling factors used, see Methods). TSS: Transcription Start Site. TES, Transcription End Site. **(D)** Logo for the most representative sequences found in the last 15 nt of snoRFs for *Rpp30* mutants (issued from *fasta* files containing all sequences).

Supplementary Figure 8 Workflow related to miRNA normalization, scale factors and global cascade annotation. Representation of the Workflows used to perform miRNA normalization **(A)** and the Scale Factors **(B)**. Shown are Scale factors for all the genotypes used in this study. For simplicity, only *white*-, *Rpp30*^18.^*^2^homoz., Rpp30^18^*^.^*^2^;ubiRpp30GFP, dTrm7_34^∗^/Tb,Sb^∗^* heterozygous, *dTrm7_34^∗^* homozygous and *dTrm7_34^∗^, dTrm7_32^∗^* double mutants have been used for the main figures. **(C)** Workflow used to generate the cascade annotations represented in Figures 2, 3, 5.

Supplementary Figure 9 Workflow related to Bam Coverages and tRNA expression heatmaps. Scheme of the Workflows used for generation of tRFs Bam Coverages represented in Figures 2, 3, 5. **(A)**, tRFs Heatmaps represented in Supplementary Figures 2, 4 **(B)**, and snoRFs Bam Coverage represented in Supplementary Figure 7 **(C)**.

Supplementary Table 1 Comparison of some existing tRFs bioinformatic analysis tools. Comparison is only based on some selected bibliographic resources available in Pubmed, April, 2020. y: yes, n: no. “-” indicates that the information is not relevant or that the item doesn’t exist.
