# Supplementary material for: tRNA Fragments Populations Analysis in Mutants Affecting tRNAs Processing and tRNA Methylation
Source: Front Genet. 2020 Oct 9;11:518949. doi: 10.3389/fgene.2020.518949 (PMC7586317; doi:10.3389/fgene.2020.518949)
Supplement: Supplementary Figure 1 — tRFs could originate from tRNA space and non-tRNA space: (A) Representation of the different genomic loci that can give rise to tRFs: tRNAs, tRNA-lookalikes, truncated tRNAs or repeated elements. (B) tRFs size distribution of reads matching “tRNA space” (gray) and “non-tRNA space” (blue). (C) Fold change of tRF reads matching the “tRNA-space” versus tRFs reads matching the “non-tRNA space.” (D) tRFs matching the “non-tRNA space” corresponding to tRFs-CCA (dark pink) and tRFs-non-CCA (light pink) in the represented genotypes. (E) comparison between Bam Coverages obtained with 15–29 nt (left, same as Figure 3B) and 17–29 nt (right) in different genotypes. [file Presentation_1.pdf]

Sup. Fig. 1

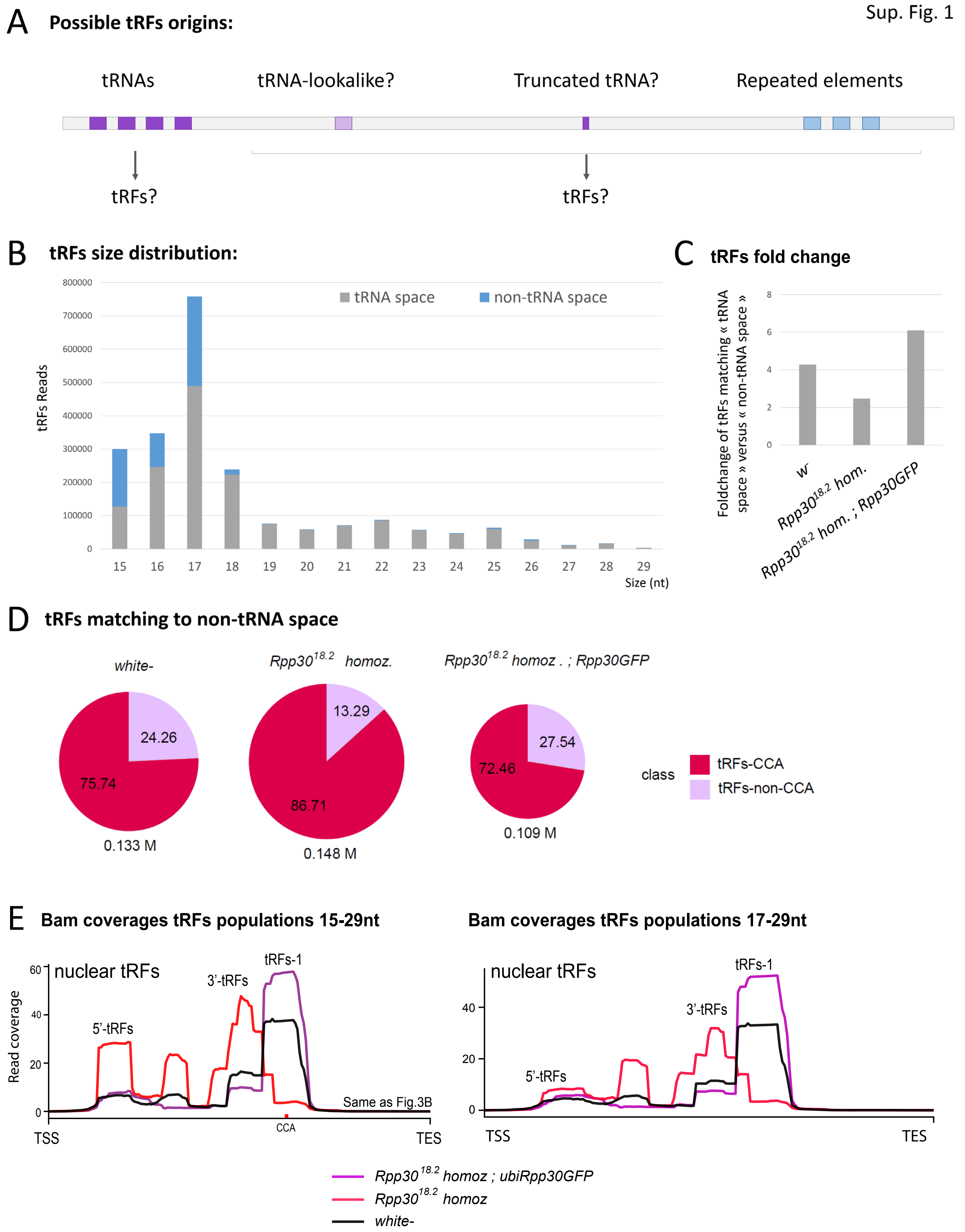
A
Possible tRFs origins:
tRNAs
tRNA-lookalike?
Truncated tRNA?
Repeated elements
tRFs?
tRFs?
B
tRFs size distribution:
tRFs Reads
800000
700000
600000
500000
400000
300000
200000
100000
0
tRNA space
non-tRNA space
15 16 17 18 19 20 21 22 23 24 25 26 27 28 29
Size (nt)
C
tRFs fold change
Foldchange of tRFs matching « tRNA space » versus « non-tRNA space »
8
6
4
2
0
w-
Rpp30 18.2 hom.
Rpp30 18.2 hom. ; Rpp30GFP
D
tRFs matching to non-tRNA space
white-
Rpp30 18.2 homoz.
Rpp30 18.2 homoz . ; Rpp30GFP
24.26
75.74
0.133 M
13.29
86.71
0.148 M
27.54
72.46
0.109 M
class
tRFs-CCA
tRFs-non-CCA
E
Bam coverages tRFs populations 15-29nt
Bam coverages tRFs populations 17-29nt
nuclear tRFs
Read coverage
60
40
20
0
5'-tRFs
3'-tRFs
tRFs-1
Same as Fig.3B
CCA
TSS
TES
nuclear tRFs
40
20
0
5'-tRFs
3'-tRFs
tRFs-1
TSS
TES
Rpp30 18.2 homoz ; ubiRpp30GFP
Rpp30 18.2 homoz
white-

Sup.Fig.2

A tRNA expression heatmap
Unique tRNA mature CCA-edited seq.

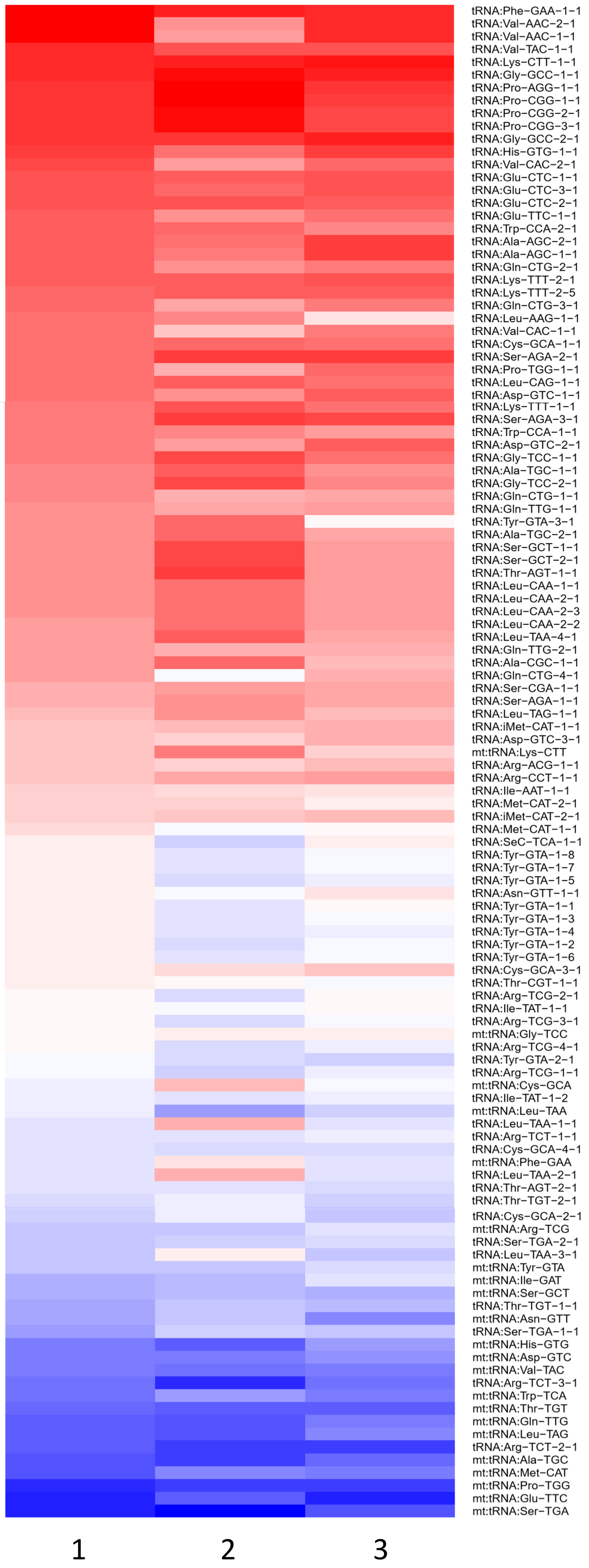

1 *White⁻*
2 *Rpp30$^{18.2}$ hom.*
3 *Rpp30$^{18.2}$ hom. ; Rpp30GFP (rescue)*

B Ratio of counts *Rpp30$^{18.2}$/w⁻*
Unique tRNA mature CCA-edited seq.

tRNA:Leu-TAA-2-1	8,96	mt:tRNA:Arg-TCG	0,95
tRNA:Leu-TAA-1-1	8,16	tRNA:Arg-TCT-1-1	0,91
mt:tRNA:Glu-TTC	7,41	tRNA:Thr-CGT-1-1	0,90
mt:tRNA:Cys-GCA	6,68	tRNA:Ile-AAT-1-1	0,84
tRNA:Thr-AGT-1-1	6,22	mt:tRNA:Thr-TGT	0,82
tRNA:Leu-TAA-3-1	5,22	tRNA:Trp-CCA-2-1	0,82
tRNA:Ser-GCT-1-1	4,59	tRNA:Ile-TAT-1-2	0,80
tRNA:Ser-GCT-2-1	4,58	mt:tRNA:Val-TAC	0,79
mt:tRNA:Lys-CTT	3,91	mt:tRNA:Gln-TTG	0,79
tRNA:Leu-TAA-4-1	3,87	mt:tRNA:Leu-TAG	0,79
tRNA:Gly-TCC-2-1	3,29	tRNA:Asp-GTC-3-1	0,78
tRNA:Gly-TCC-1-1	3,21	tRNA:Ile-TAT-1-1	0,77
tRNA:Ser-AGA-3-1	3,20	tRNA:Trp-CCA-1-1	0,75
tRNA:Ala-CGC-1-1	3,17	tRNA:Arg-ACG-1-1	0,75
tRNA:Ser-AGA-2-1	3,13	tRNA:Cys-GCA-4-1	0,72
mt:tRNA:Met-CAT	3,09	tRNA:Asn-GTT-1-1	0,68
tRNA:Pro-CGG-1-1	3,06	tRNA:Glu-CTC-1-1	0,66
mt:tRNA:Phe-GAA	3,00	tRNA:Gln-TTG-2-1	0,65
tRNA:Pro-AGG-1-1	2,98	tRNA:Glu-CTC-3-1	0,64
tRNA:Pro-CGG-3-1	2,82	mt:tRNA:Ala-TGC	0,62
tRNA:Pro-CGG-2-1	2,78	tRNA:Ala-AGC-2-1	0,59
mt:tRNA:Trp-TCA	2,69	tRNA:Ala-AGC-1-1	0,57
tRNA:Ser-TGA-1-1	2,51	tRNA:Gln-TTG-1-1	0,56
tRNA:Leu-TAG-1-1	2,36	tRNA:Tyr-GTA-2-1	0,54
tRNA:Tyr-GTA-3-1	2,36	tRNA:Leu-AAG-1-1	0,54
tRNA:Leu-CAA-2-2	2,27	tRNA:Arg-TCT-2-1	0,53
tRNA:Gly-GCC-1-1	2,26	tRNA:Asp-GTC-1-1	0,52
tRNA:Lys-TTT-1-1	2,24	tRNA:Asp-GTC-2-1	0,51
tRNA:Leu-CAA-2-3	2,24	tRNA:Arg-TCG-4-1	0,50
tRNA:Ala-TGC-1-1	2,23	tRNA:Met-CAT-1-1	0,49
tRNA:Leu-CAA-2-1	2,22	tRNA:Phe-GAA-1-1	0,49
tRNA:Leu-CAA-1-1	2,20	tRNA:Tyr-GTA-1-6	0,48
tRNA:Ala-TGC-2-1	2,19	tRNA:Val-TAC-1-1	0,46
tRNA:Thr-TGT-1-1	1,99	tRNA:Gln-CTG-1-1	0,45
mt:tRNA:Asn-GTT	1,95	tRNA:Tyr-GTA-1-4	0,44
tRNA:Arg-CCT-1-1	1,93	tRNA:Arg-TCG-2-1	0,44
mt:tRNA:Pro-TGG	1,85	mt:tRNA:His-GTG	0,44
tRNA:Ser-AGA-1-1	1,66	tRNA:Arg-TCG-1-1	0,43
tRNA:Cys-GCA-2-1	1,64	tRNA:Tyr-GTA-1-3	0,43
tRNA:Ser-CGA-1-1	1,57	tRNA:Tyr-GTA-1-7	0,43
tRNA:Cys-GCA-3-1	1,56	tRNA:Arg-TCG-3-1	0,43
tRNA:Leu-CAG-1-1	1,55	tRNA:Tyr-GTA-1-8	0,42
tRNA:iMet-CAT-2-1	1,41	tRNA:Tyr-GTA-1-1	0,40
tRNA:Thr-TGT-2-1	1,39	tRNA:His-GTG-1-1	0,39
tRNA:Lys-CTT-1-1	1,29	tRNA:Tyr-GTA-1-2	0,37
mt:tRNA:Ile-GAT	1,27	tRNA:Gln-CTG-2-1	0,35
mt:tRNA:Gly-TCC	1,27	tRNA:Tyr-GTA-1-5	0,35
tRNA:Cys-GCA-1-1	1,24	tRNA:Glu-TTC-1-1	0,34
tRNA:Ser-TGA-2-1	1,21	tRNA:Pro-TGG-1-1	0,30
mt:tRNA:Asp-GTC	1,19	tRNA:SeC-TCA-1-1	0,28
tRNA:Lys-TTT-2-5	1,17	tRNA:Gln-CTG-3-1	0,26
mt:tRNA:Ser-GCT	1,16	tRNA:Val-CAC-2-1	0,20
tRNA:iMet-CAT-1-1	1,15	mt:tRNA:Leu-TAA	0,18
tRNA:Glu-CTC-2-1	1,10	tRNA:Arg-TCT-3-1	0,15
mt:tRNA:Tyr-GTA	1,10	tRNA:Val-CAC-1-1	0,15
tRNA:Lys-TTT-2-1	1,07	tRNA:Gln-CTG-4-1	0,12
tRNA:Thr-AGT-2-1	1,07	tRNA:Val-AAC-2-1	0,05
tRNA:Gly-GCC-2-1	1,00	tRNA:Val-AAC-1-1	0,04
tRNA:Met-CAT-2-1	0,97	mt:tRNA:Ser-TGA	0,00

C tRNA expression heatmap
tRNA extended CCA-edited

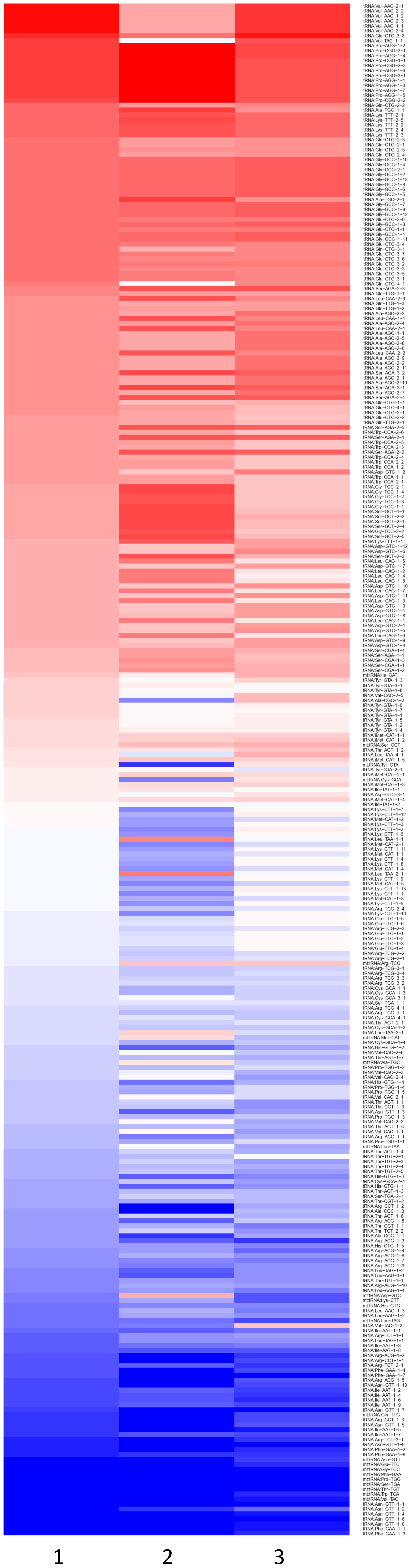

Profiles of highly expressed tRFs in control *white-* ovaries compared to *Rpp30* mutant:

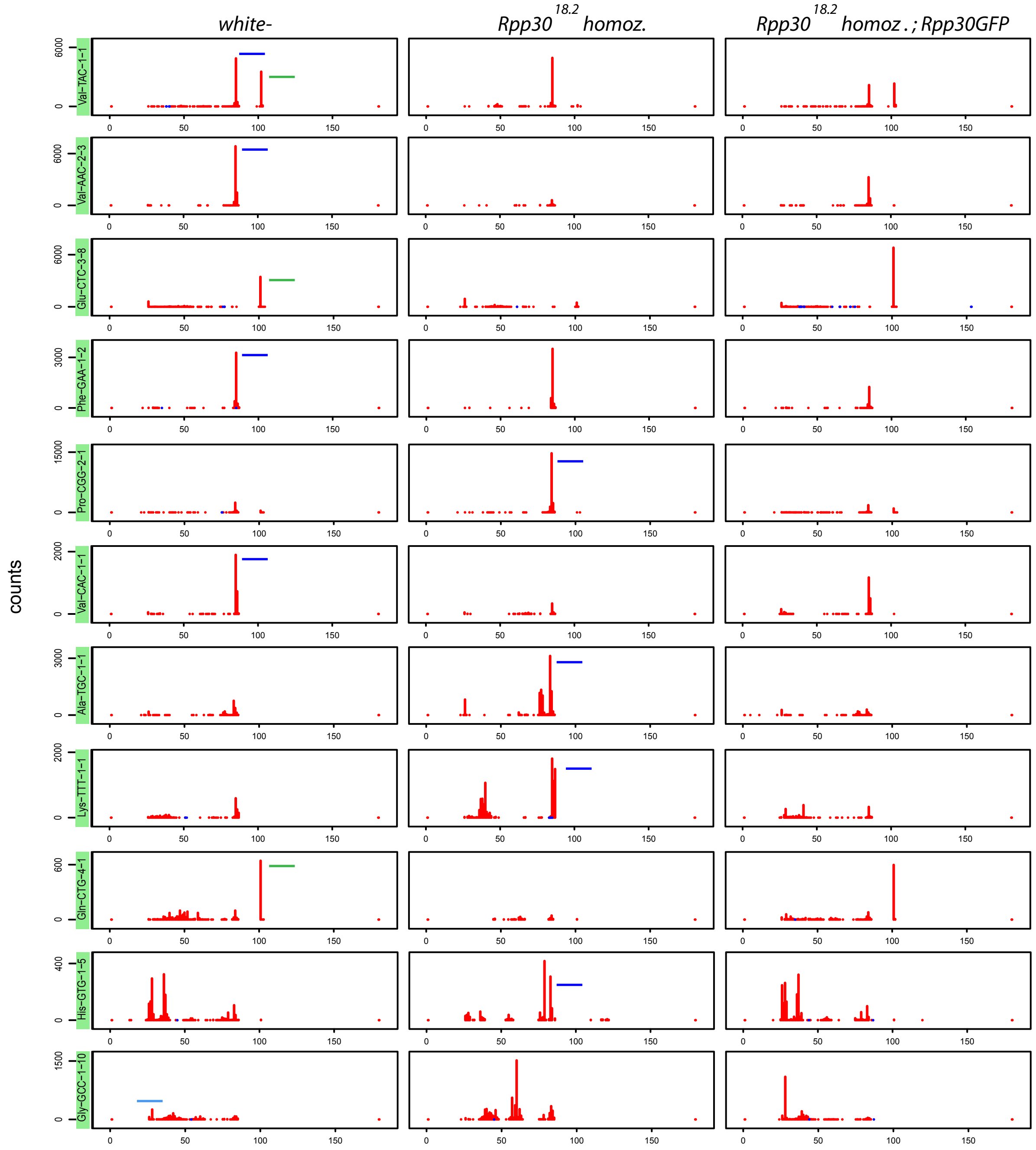

Fig.Sup.4

A Heatmap tRNA expression:

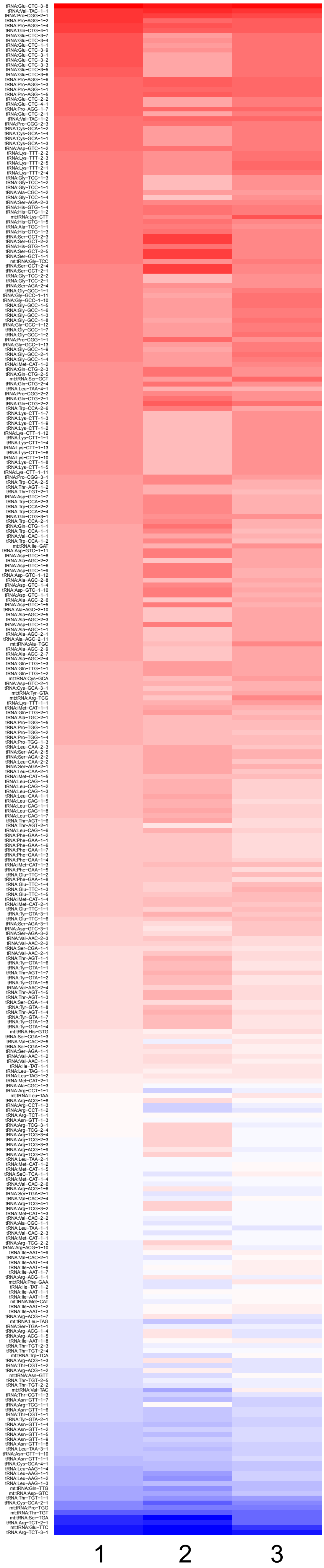

B Ratio *dTrm7_34** homoz./heteroz.:

mt:tRNA:Glu-TTC	0.00	mt:tRNA:Ile-GAT	0.75	tRNA:iMet-CAT-1-4	1.33
mt:tRNA:Ser-TGA	0.00	mt:tRNA:His-GTG	0.75	tRNA:Val-AAC-1-2	1.34
tRNA:Arg-TCT-3-1	0.00	tRNA:Val-CAC-2-6	0.75	tRNA:Gln-CTG-3-1	1.34
tRNA:Glu-CTC-3-1	0.21	mt:tRNA:Trp-TCA	0.76	tRNA:Gln-TTG-1-3	1.34
tRNA:Glu-CTC-3-6	0.22	tRNA:Asn-GTT-1-4	0.77	tRNA:Leu-CAG-1-1	1.34
tRNA:Glu-CTC-3-5	0.22	tRNA:Leu-TAA-3-1	0.78	tRNA:Trp-CCA-2-6	1.34
tRNA:Glu-CTC-3-2	0.22	mt:tRNA:Cys-GCA	0.79	tRNA:His-GTG-1-2	1.34
tRNA:Glu-CTC-3-3	0.22	tRNA:Val-CAC-2-4	0.79	tRNA:Gln-TTG-1-2	1.35
tRNA:Glu-CTC-3-7	0.23	mt:tRNA:Ala-TGC	0.79	tRNA:Ile-AAT-1-5	1.36
tRNA:Glu-CTC-1-1	0.24	tRNA:Ser-AGA-3-1	0.80	tRNA:Gln-TTG-1-1	1.38
tRNA:Gly-TCC-1-4	0.24	mt:tRNA:Pro-TGG	0.80	tRNA:Ile-AAT-1-1	1.39
tRNA:Gly-TCC-1-2	0.25	tRNA:Val-CAC-2-1	0.81	tRNA:His-GTG-1-3	1.40
tRNA:Gly-TCC-1-1	0.25	mt:tRNA:Phe-GAA	0.81	tRNA:Leu-CAA-2-3	1.40
tRNA:Gly-TCC-1-3	0.26	tRNA:Asn-GTT-1-5	0.81	tRNA:Thr-TGT-2-2	1.41
tRNA:Glu-CTC-2-1	0.27	tRNA:Thr-CGT-1-2	0.82	tRNA:Pro-CGG-3-1	1.41
tRNA:Glu-CTC-3-9	0.27	tRNA:Asn-GTT-1-3	0.83	tRNA:Val-AAC-2-1	1.41
tRNA:Glu-CTC-3-4	0.27	tRNA:Val-CAC-2-2	0.83	tRNA:His-GTG-1-1	1.42
tRNA:Glu-CTC-2-2	0.28	tRNA:Ser-AGA-2-4	0.83	tRNA:Leu-CAG-1-3	1.42
tRNA:Glu-CTC-4-1	0.29	tRNA:Pro-CGG-2-1	0.83	tRNA:Leu-CAG-1-5	1.42
tRNA:Arg-CCT-1-1	0.30	tRNA:Glu-TTC-1-4	0.84	tRNA:Thr-TGT-2-4	1.42
tRNA:Arg-CCT-1-3	0.31	tRNA:Ile-TAT-1-2	0.85	tRNA:Leu-CAG-1-7	1.42
tRNA:Gly-TCC-2-2	0.35	mt:tRNA:Asp-GTC	0.85	tRNA:Ile-AAT-1-6	1.43
tRNA:Gly-TCC-2-1	0.36	mt:tRNA:Thr-TGT	0.85	tRNA:Leu-CAG-1-4	1.45
tRNA:Arg-CCT-1-2	0.36	tRNA:Ser-CGA-1-1	0.86	tRNA:Leu-CAG-1-2	1.46
tRNA:Cys-GCA-1-2	0.37	tRNA:Tyr-GTA-2-1	0.86	tRNA:Ser-AGA-2-2	1.47
tRNA:Cys-GCA-1-4	0.37	tRNA:Val-CAC-1-1	0.87	tRNA:Trp-CCA-2-5	1.48
tRNA:Cys-GCA-1-3	0.38	tRNA:Ser-AGA-3-2	0.87	tRNA:Leu-AAG-1-3	1.49
tRNA:Cys-GCA-1-1	0.38	tRNA:Leu-TAG-1-1	0.88	tRNA:Gln-TTG-2-1	1.50
tRNA:Pro-AGG-1-2	0.38	tRNA:Cys-GCA-4-1	0.89	tRNA:Leu-CAG-1-6	1.50
tRNA:Val-CAC-2-5	0.40	tRNA:Ser-CGA-1-4	0.90	tRNA:Ser-AGA-2-5	1.50
tRNA:Cys-GCA-2-1	0.41	tRNA:Glu-TTC-1-3	0.90	tRNA:Leu-CAA-2-2	1.50
mt:tRNA:Val-TAC	0.42	tRNA:Asn-GTT-1-6	0.90	tRNA:Trp-CCA-2-3	1.56
tRNA:Lys-CTT-1-2	0.42	mt:tRNA:Lys-CTT	0.91	tRNA:Ile-AAT-1-3	1.56
tRNA:Lys-CTT-1-3	0.44	tRNA:Glu-TTC-1-1	0.92	tRNA:Leu-CAG-1-8	1.57
tRNA:Lys-CTT-1-10	0.45	tRNA:Pro-TGG-1-5	0.92	tRNA:Ile-AAT-1-2	1.57
tRNA:Lys-CTT-1-7	0.45	tRNA:Met-CAT-1-3	0.92	tRNA:Leu-CAA-2-1	1.58
tRNA:Lys-CTT-1-9	0.45	mt:tRNA:Tyr-GTA	0.92	tRNA:Ser-AGA-2-1	1.59
tRNA:Lys-CTT-1-1	0.45	tRNA:Thr-CGT-1-3	0.93	tRNA:Trp-CCA-2-4	1.60
tRNA:Lys-CTT-1-8	0.45	tRNA:Ile-TAT-1-1	0.93	tRNA:Trp-CCA-2-2	1.61
tRNA:Lys-CTT-1-12	0.46	tRNA:Pro-TGG-1-3	0.93	tRNA:Ile-AAT-1-8	1.64
tRNA:Lys-CTT-1-6	0.46	tRNA:Glu-TTC-1-2	0.93	tRNA:Leu-CAA-1-1	1.64
tRNA:Lys-CTT-1-4	0.46	tRNA:Pro-TGG-1-4	0.94	tRNA:Trp-CCA-2-1	1.65
tRNA:Lys-CTT-1-13	0.47	tRNA:Glu-TTC-1-5	0.94	tRNA:Asp-GTC-1-7	1.68
tRNA:Lys-CTT-1-11	0.48	tRNA:Pro-TGG-1-2	0.94	tRNA:Trp-CCA-1-1	1.70
tRNA:Lys-CTT-1-5	0.48	tRNA:Ala-TGC-2-1	0.94	tRNA:Trp-CCA-1-2	1.71
mt:tRNA:Leu-TAG	0.49	mt:tRNA:Leu-TAA	0.95	tRNA:Arg-ACG-1-8	1.72
tRNA:Thr-AGT-2-1	0.50	tRNA:Met-CAT-1-2	0.95	tRNA:Tyr-GTA-3-1	1.76
tRNA:Ala-AGC-2-2	0.50	tRNA:Ser-AGA-1-1	0.95	tRNA:Thr-TGT-2-5	1.77
tRNA:Ala-CGC-1-2	0.51	tRNA:Pro-TGG-1-1	0.96	tRNA:Tyr-GTA-1-2	1.80
tRNA:Leu-TAA-4-1	0.53	mt:tRNA:Met-CAT	0.96	tRNA:Tyr-GTA-1-3	1.80
tRNA:Ala-AGC-2-10	0.54	tRNA:Leu-TAA-2-1	0.97	tRNA:Tyr-GTA-1-4	1.81
tRNA:Ala-AGC-2-3	0.54	tRNA:Pro-CGG-2-3	0.97	tRNA:Arg-ACG-1-9	1.83
tRNA:Ala-AGC-2-9	0.54	mt:tRNA:Asn-GTT	0.98	tRNA:Tyr-GTA-1-6	1.83
tRNA:Ala-AGC-2-5	0.54	tRNA:Met-CAT-2-1	0.99	tRNA:Gln-CTG-2-1	1.86
tRNA:Ala-AGC-2-1	0.55	tRNA:Asp-GTC-2-1	1.00	tRNA:Gln-CTG-2-5	1.87
tRNA:Ala-AGC-2-4	0.56	tRNA:Phe-GAA-1-7	1.00	tRNA:Tyr-GTA-1-1	1.87
tRNA:Gln-CTG-4-1	0.56	tRNA:Met-CAT-1-5	1.01	tRNA:Gln-CTG-2-4	1.90
tRNA:Ala-AGC-1-1	0.57	tRNA:Phe-GAA-1-2	1.01	tRNA:Thr-AGT-1-6	1.90
tRNA:Ala-AGC-2-6	0.58	tRNA:Asp-GTC-1-2	1.02	tRNA:Tyr-GTA-1-5	1.90
tRNA:Gly-GCC-1-8	0.59	tRNA:Glu-TTC-1-6	1.02	tRNA:Arg-ACG-1-6	1.92
tRNA:Ala-AGC-2-7	0.59	tRNA:Ser-CGA-1-3	1.02	tRNA:Tyr-GTA-1-7	1.94
tRNA:Gly-GCC-1-11	0.59	tRNA:Arg-TCT-1-1	1.02	tRNA:Gln-CTG-2-3	1.94
tRNA:Leu-AAG-1-2	0.59	tRNA:Phe-GAA-1-1	1.03	tRNA:Tyr-GTA-1-8	1.96
tRNA:SeC-TCA-1-1	0.59	tRNA:Lys-TTT-1-1	1.03	tRNA:Pro-CGG-1-1	1.97
tRNA:Ala-AGC-2-11	0.59	tRNA:Phe-GAA-1-3	1.04	tRNA:Asp-GTC-1-8	2.07
tRNA:Ala-AGC-2-8	0.60	tRNA:Phe-GAA-1-6	1.04	tRNA:Thr-AGT-1-1	2.07
tRNA:Lys-TTT-2-5	0.60	tRNA:Val-TAC-1-2	1.04	tRNA:Asp-GTC-1-4	2.09
tRNA:Gly-GCC-1-6	0.60	tRNA:Ser-CGA-1-2	1.04	tRNA:Asp-GTC-1-11	2.12
tRNA:Gly-GCC-1-1	0.61	tRNA:Ala-TGC-1-1	1.05	tRNA:Arg-ACG-1-10	2.12
tRNA:Cys-GCA-3-1	0.61	tRNA:Met-CAT-1-4	1.05	tRNA:Asp-GTC-1-9	2.12
tRNA:Glu-CTC-3-8	0.61	tRNA:Phe-GAA-1-4	1.05	tRNA:Asp-GTC-1-6	2.13
tRNA:Lys-TTT-2-2	0.61	tRNA:Phe-GAA-1-5	1.08	tRNA:Asp-GTC-1-12	2.13
tRNA:Gly-GCC-1-5	0.62	tRNA:Pro-AGG-1-6	1.08	tRNA:Thr-AGT-1-3	2.15
tRNA:Gly-GCC-1-7	0.62	tRNA:Pro-AGG-1-1	1.09	tRNA:Thr-AGT-1-5	2.16
tRNA:Gly-GCC-1-9	0.62	tRNA:iMet-CAT-1-5	1.09	tRNA:Asp-GTC-1-1	2.17
tRNA:Lys-TTT-2-1	0.62	tRNA:Asn-GTT-1-1	1.10	tRNA:Asp-GTC-1-5	2.18
tRNA:Gly-GCC-1-13	0.62	tRNA:Pro-AGG-1-3	1.10	tRNA:Arg-ACG-1-1	2.21
tRNA:Gly-GCC-1-10	0.63	tRNA:Pro-AGG-1-7	1.11	tRNA:Thr-AGT-1-7	2.23
tRNA:Lys-TTT-2-3	0.63	tRNA:Asn-GTT-1-8	1.12	tRNA:Asp-GTC-1-10	2.25
mt:tRNA:Gln-TTG	0.63	tRNA:Asn-GTT-1-2	1.12	tRNA:Thr-AGT-1-4	2.28
tRNA:Gly-GCC-1-3	0.64	tRNA:Phe-GAA-1-8	1.13	tRNA:Asp-GTC-1-3	2.29
tRNA:Gly-GCC-2-1	0.64	tRNA:Ser-TGA-1-1	1.13	tRNA:Gln-CTG-1-1	2.42
tRNA:Gly-GCC-1-12	0.65	tRNA:Pro-AGG-1-5	1.13	tRNA:Arg-TCG-3-1	2.56
tRNA:Gly-GCC-1-2	0.65	tRNA:Met-CAT-1-1	1.14	tRNA:Arg-TCG-3-3	2.60
tRNA:Lys-TTT-2-4	0.66	tRNA:Leu-TAA-1-1	1.14	tRNA:Arg-ACG-1-2	2.62
tRNA:Asn-GTT-1-7	0.66	tRNA:Val-TAC-1-1	1.14	tRNA:Arg-ACG-1-5	2.62
tRNA:Asp-GTC-3-1	0.66	tRNA:Val-AAC-2-2	1.14	tRNA:Arg-ACG-1-7	2.67
tRNA:Thr-TGT-1-1	0.66	tRNA:Asn-GTT-1-10	1.15	tRNA:Arg-TCG-2-4	2.68
tRNA:Gly-GCC-1-4	0.66	tRNA:Leu-AAG-1-4	1.17	tRNA:Arg-TCG-2-1	2.68
tRNA:Leu-AAG-1-1	0.67	tRNA:Asn-GTT-1-9	1.21	tRNA:Arg-TCG-3-4	2.71
tRNA:Ala-CGC-1-3	0.67	tRNA:Ile-AAT-1-7	1.21	tRNA:Arg-TCG-2-3	2.71
tRNA:Ala-CGC-1-1	0.67	tRNA:Thr-TGT-2-3	1.23	tRNA:Arg-ACG-1-4	2.84
tRNA:Thr-AGT-1-2	0.67	tRNA:Ile-AAT-1-4	1.24	tRNA:Gln-CTG-2-2	2.95
mt:tRNA:Arg-TCG	0.67	tRNA:Val-AAC-1-1	1.24	tRNA:Arg-TCG-4-1	2.99
tRNA:Thr-TGT-2-1	0.68	tRNA:iMet-CAT-1-1	1.28	tRNA:Arg-TCG-3-2	3.02
tRNA:Arg-TCT-2-1	0.68	tRNA:Val-AAC-2-4	1.28	tRNA:Arg-ACG-1-3	3.12
tRNA:Ser-TGA-2-1	0.69	tRNA:Leu-TAG-1-2	1.28	tRNA:Arg-TCG-1-1	3.25
tRNA:iMet-CAT-1-2	0.70	tRNA:iMet-CAT-1-3	1.29	tRNA:Arg-TCG-2-2	3.27
tRNA:Pro-AGG-1-4	0.72	tRNA:Pro-CGG-2-2	1.30	tRNA:Ser-GCT-2-2	3.60
mt:tRNA:Ser-GCT	0.72	tRNA:His-GTG-1-5	1.31	tRNA:Ser-GCT-2-3	3.60
mt:tRNA:Gly-TCC	0.72	tRNA:Ile-AAT-1-9	1.31	tRNA:Ser-GCT-2-4	3.65
tRNA:Val-CAC-2-3	0.73	tRNA:His-GTG-1-4	1.31	tRNA:Ser-GCT-2-5	3.70
tRNA:Ser-AGA-2-3	0.73	tRNA:Val-AAC-2-3	1.31	tRNA:Ser-GCT-1-1	3.75
tRNA:Thr-CGT-1-1	0.75	tRNA:iMet-CAT-2-1	1.32	tRNA:Ser-GCT-2-1	3.81

1. dTrm7_34*/TbSb

2. dTrm7_34*hom.

3. dTrm7_34*, dTrm7_32*hom.

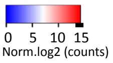

Sup. Fig. 5

Profiles of highly expressed tRFs in control ovaries compared to tRNA methylation mutants:

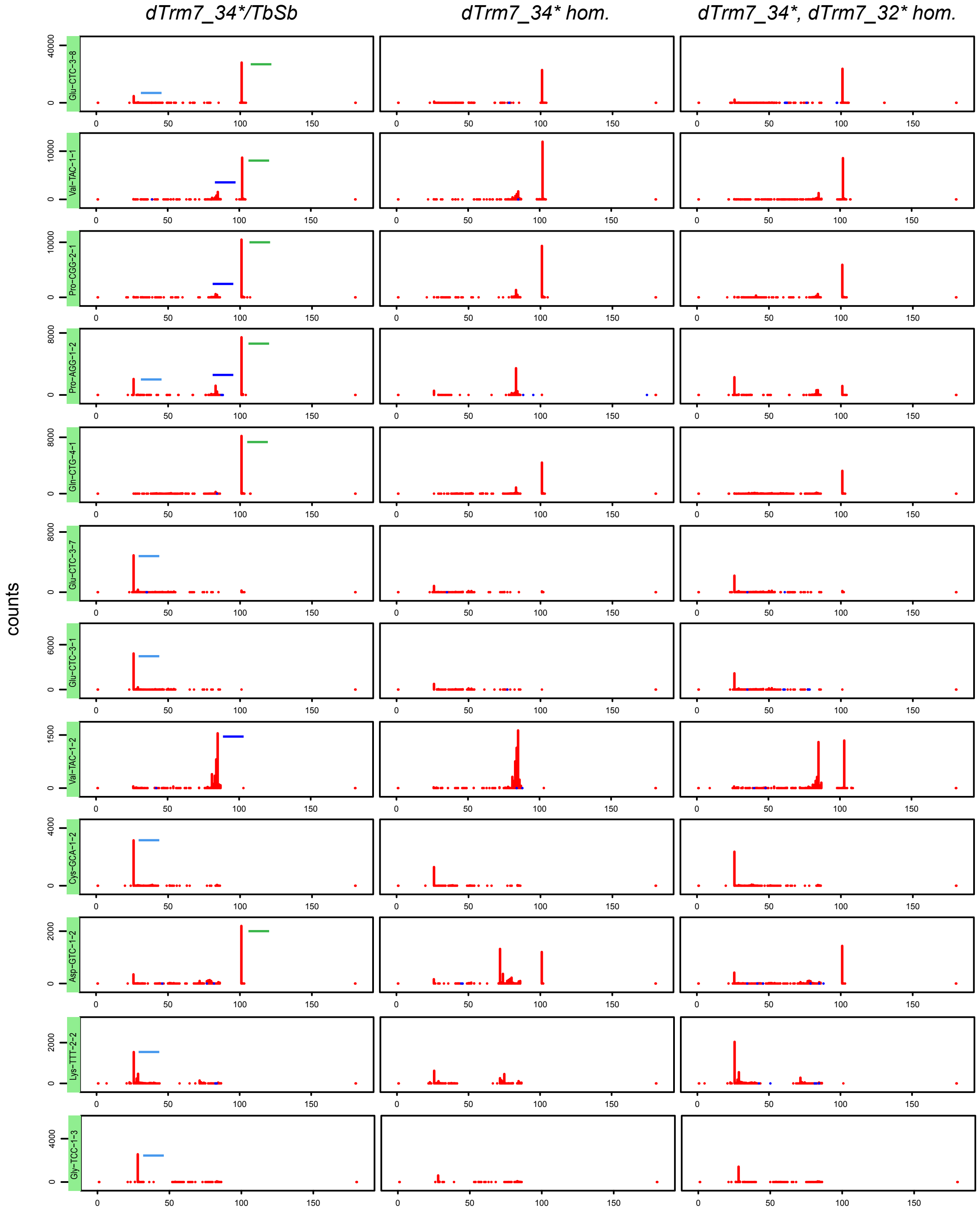

Sup. Fig.6

tRFs profiles of tRNAs methylated by dTrm7_34 and dTrm7_32:

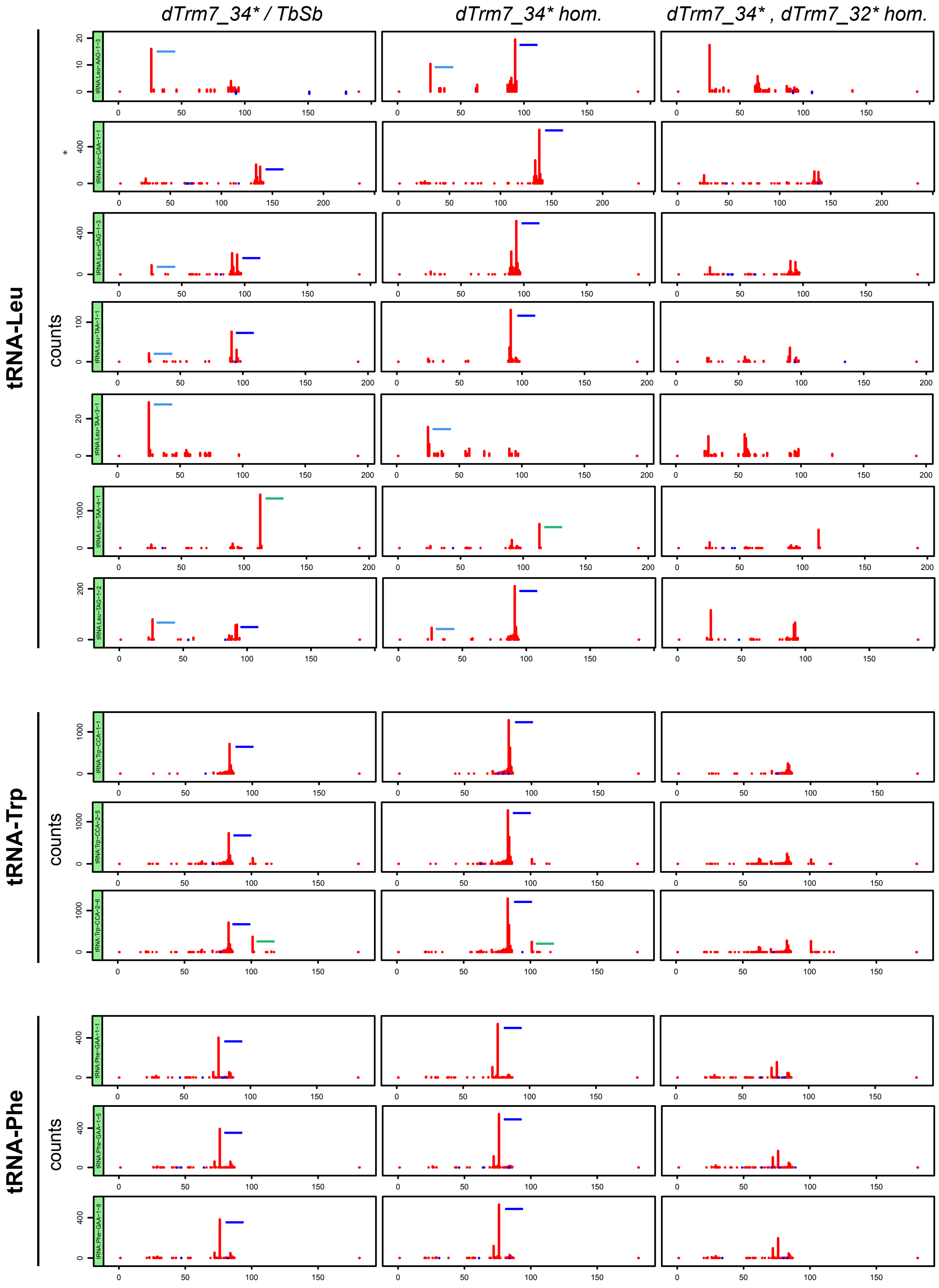

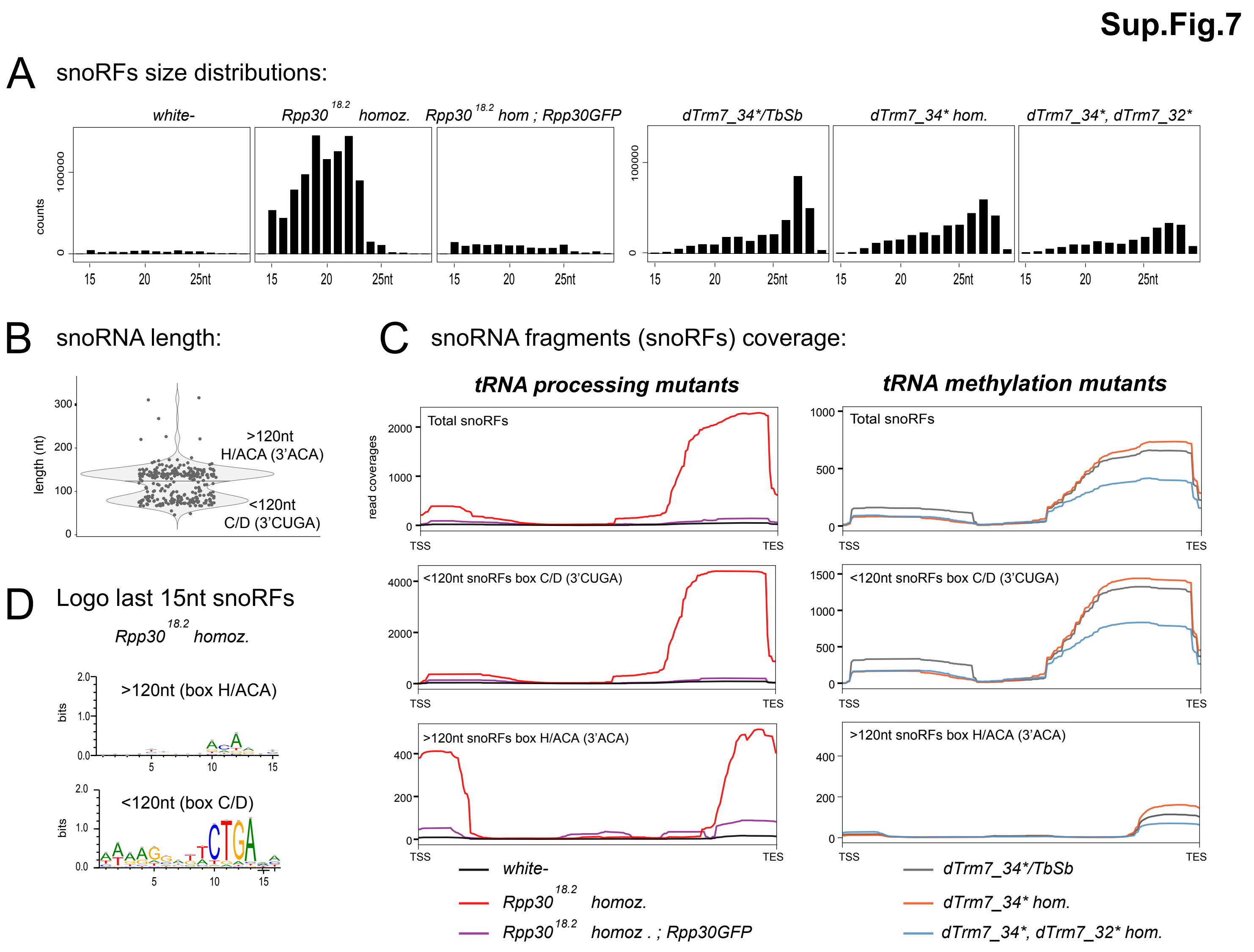
Sup.Fig.7
A
snoRFs size distributions:
white-
Rpp30 18.2 homoz.
Rpp30 18.2 hom ; Rpp30GFP
dTrm7_34*/TbSb
dTrm7_34* hom.
dTrm7_34*, dTrm7_32*
counts
100000
0
15
20
25nt
B
snoRNA length:
length (nt)
300
200
100
0
>120nt
H/ACA (3'ACA)
<120nt
C/D (3'CUGA)
C
snoRNA fragments (snoRFs) coverage:
tRNA processing mutants
tRNA methylation mutants
read coverages
Total snoRFs
<120nt snoRFs box C/D (3'CUGA)
>120nt snoRFs box H/ACA (3'ACA)
TSS
TES
white-
Rpp30 18.2 homoz.
Rpp30 18.2 homoz . ; Rpp30GFP
dTrm7_34*/TbSb
dTrm7_34* hom.
dTrm7_34*, dTrm7_32* hom.
D
Logo last 15nt snoRFs
Rpp30 18.2 homoz.
>120nt (box H/ACA)
<120nt (box C/D)
bits
2.0
1.0
0.0
5
10
15

Sup.Fig.8

A

miRNA normalization:

small RNA reads input fasta files

miRcounts Counts miRNA alignments from small RNAsequence data (Galaxy Version 1.3.2)

Column Join on Collections (Galaxy Version 0.0.3) of miRcounts tabular files

DESeq2 normalization of hit lists (geometric method) (Galaxy Version 1.0.1)

Compute an expression on every row (Galaxy Version 1.2.0)

B

normalization factors:

**Genotype**	**NF= Norm. factors**	**1/NF**
Rpp30 $^{18.2}$, mnk ^{P6} homoz	0.50	**2.00**
Rpp30 $^{18.2}$ homoz. ; Rpp30GFP	2.39	**0.42**
Rpp30 $^{18.2}$ / Rpp30 ^{PE}	1.23	**0.81**
Rpp30 $^{18.2}$ homoz.	0.43	**2.33**
w-	1.76	**0.57**
dTrm7_34 / TbSb*	1.26	**0.80**
dTrm7_34 homoz.*	0.77	**1.30**
dTrm7_34 , dTrm7_32* hom.*	1.89	**0.53**
dTrm7_34 / Def9487*	0.56	**1.80**

C

Annotate datasets:

small RNA reads input fasta files depeleted from rRNA.

Annotate smRNA dataset by iterative alignments with sRbowtie
(Galaxy Version 2.4.0).
0 mismatch allowed.
Alignment steps: in the following order tRNA, tRNA-CCA-edited, miRNA, TE-derived, all-ncRNA, all genes, all intergenic.
Generation of a cascade annotation analysis and a barplot.

Sup.Fig.9

A

tRNA fragments (tRFs) Bam Coverage:

tRFs input fasta files

sRBowtie (for small RNA short reads (Galaxy Version 2.1.1)
Matched on DNA, multiple mappers, randomly matched at a single position
0MM
Ref: tRNA-CCA-edited-extended

BamCoverage generates a coverage bigWig file from a given BAM or CRAM file (Galaxy Version 3.1.2.0.0)
Scale factors were used for each genotype.

ComputeMatrix prepares data for plotting a heatmap or a profile of given regions (Galaxy Version 3.1.2.0.0)
Regions to plot: Nuclear tRNAs (all, 5′ or 3′) or Mitoch. tRNAs Bed files.

plotProfile creates a profile plot for score distributions across genomic regions (Galaxy Version 3.1.2.0.0)

B

tRF Expression workflow:

tRF collection list

sRBowtie (for small RNA short reads (Galaxy Version 2.1.1)
Matched on DNA, multiple mappers, randomly matched at a single position
0MM
Ref: tRNA-CCA-edited

Parse items in sRbowtie alignment (Galaxy Version 1.0.6)

DESeq2 normalization of hit lists (geometric method) (Galaxy Version 1.0.1)

Cut columns from a table (Galaxy Version 1.0.2)

Sort data in ascending or descending order (Galaxy Version 1.1.1)

Plot heatmap with high number of rows (Galaxy Version 1.0.0).
Data transformation: Log2(value+1)

Cut columns from a table (Galaxy Version 1.0.2)

Compute an expression on every row (Galaxy Version 1.2.0)

C

snoRNA fragment (snoRF) Bam Coverage:

snoRNA input fasta files

sRBowtie (for small RNA short reads (Galaxy Version 2.1.1)
Matched on DNA, multiple mappers, randomly matched at a single position
0MM
Ref: snoRNA

BamCoverage generates a coverage bigWig file from a given BAM or CRAM file (Galaxy Version 3.1.2.0.0)
Scale factors were used for different genotypes.

ComputeMatrix prepares data for plotting a heatmap or a profile of given regions (Galaxy Version 3.1.2.0.0)
Regions to plot:
snoRNA Bed description file, or snoRNA>120nt or snoRNA<120nt.

plotProfile creates a profile plot for score distributions across genomic regions (Galaxy Version 3.1.2.0.0)
